# Supplementary material for: Direct interaction between RSV polymerase L and active Rab11a mediates viral ribonucleoprotein transport to assembly sites
Source: PLoS Pathog. 2026 Aug 3;22(8):e1013671. doi: 10.1371/journal.ppat.1013671 (PMC13456277; doi:10.1371/journal.ppat.1013671)
Supplement: Supplementary file 1 [file ppat.1013671.s016.pdf]

Dear editorial team,

Thank you for your evaluation of our manuscript and the acknowledgment that we have substantially improved our work following two rounds of reviewer's and editor's comments.

We are fully aware that many questions remain regarding the precise mechanisms of RNP export in association with Rab11a. However, our article precisely characterizes the interaction between active Rab11a and the polymerase, with data from cellular assays and purified proteins that are fully consistent. Receiving yet another wave of new questions at this stage feels discouraging.

We do hope that our response below will clarify this additional point and the minor comments. We would also be happy to discuss any additional points through a video call at your earliest convenience, should you require it.

Best Regards,

Additional Editor Comments (if provided):

Dear Dr. Rameix-Welti,

Thank you for submitting your revised manuscript to *PLOS Pathogens*. The editorial team has carefully evaluated the revised version and agrees that the manuscript has been substantially improved following the reviewers' comments.

However, the editorial team would like to raise **one additional point** that was not addressed during peer review. Despite being an unusual request, the editorial team considers that an answer to these concerns would substantially improve the overall understanding of role of Rab11a in RSV infection. We hope the authors agree with us.

In Figures 1A and 1C, the different viral proteins are analysed under non-equivalent experimental conditions. For consistency, these conditions should be normalized.

Specifically, we believe it is important to repeat the experiments in Figure 3 to include the P+L, P+N, P+M2-1 and P+M conditions to demonstrate that Rab11a does not relocalize to the condensates (or other structures) formed under these conditions. This control is particularly important because P+N and P+M2-1 have previously been shown to undergo phase separation, and the present study suggests that P+L may also form condensates/aggregates. It is therefore important to distinguish whether Rab11a recruitment is specific to the L-containing complex or simply a consequence of condensate formation.

Regarding the inconsistency between the immunoprecipitation and immunofluorescence experiments presented in Figure 1, it should be noted that these are different experiments in different experimental systems. The important point is that the results are consistent across the experiments and systems.

Figure 3 concerns the study of Rab11a-L interactions in vitro with purified proteins. It is not feasible to repeat all of these experiments by purifying N, P, M2-1, and M. That would go far beyond the scope of this paper.

If the question is whether Rab11 is recruited to the condensates formed by N and P (Fig 1C ?), we have verified that Rab11 is not recruited to the VFs in the absence of L: N+P; N+P+M2, N+P+M. In contrast, Rab11a is recruited at the periphery of the VRs in the presence o f L (see plot profile in conditions N+P+L). This rules out a non-specific recruitment of Rab11a in liquid condensates.

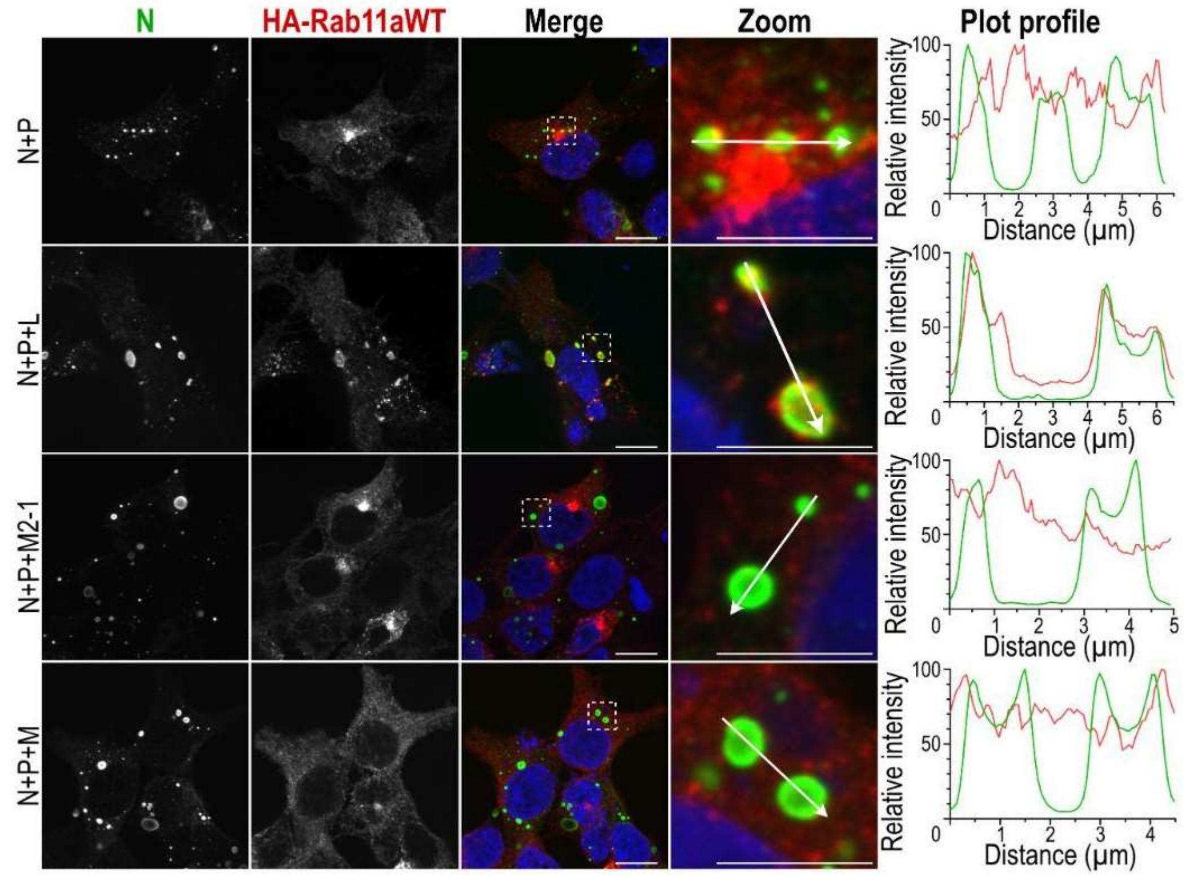

Fig legend : Recruitment of Rab11a to pseudo-viral factories in the presence of individual viral proteins. HEK-293T cells were co-transfected with HA-Rab11a, the N and P proteins (which form the pseudo-VF), and one of the following viral proteins: L, M2-1, or M. After 24 hours, the cells were fixed, immunolabeled, and then observed by confocal microscopy. Representative images showing the pseudo-VFs (N staining, green), HA-Rab11a (red), and nuclei (blue, Hoechst). Scale bars: 10 µm (main images), 5 µm (magnified views). Representative images from three independent experiments.

In addition, please clarify why the manuscript refers to the P+L structures as "aggregates" rather than phase-separated condensates. Finally, the Discussion currently suggests that the P+L aggregates are likely to be an artifact. However, the fact that they do not happen in infection does not mean that they may not happen in this transfection system. A plausible alternative explanation is that they arise from the experimental conditions, such as the high protein concentrations achieved in the transient transfection system, which may not faithfully mimic viral infection or that somehow N restricts the entrance of Rab11a into IB from RSV infected cells. Please comment on this possibility.

Regarding the aggregates observed during transient expression of L and P and Rab11a, we considered them to be artifacts since they do not exist in infected cells. Consequently, we did not attempt to characterize these structures but used them solely as a marker of interaction to complement the co-immunoprecipitation experiments. Investigating their intrinsic biophysical

properties may be interesting indeed, however it would be way beyond the scope of our study. As their irregular geometric shape suggests aggregates at first sight, we believe it is fair to categorize them this way in this study.

We propose to clarify this point in the discussion (p. 43): "Surprisingly, L-GFP **forms** aggregates**, so termed because of their irregular shape,** when expressed in the presence of Rab11a overexpression."

As for the mechanism of their formation, we can only speculate, and it is clear that our conditions are far from physiological, since we have Rab11 overexpression and L and P expression in the absence of N-and thus without the formation of viral condensates (such as viral factories). Our hypothesis is that in the infected cell, L polymerase is recruited into the viral factories. Active Rab11a is bound to the vesicles, and a portion of the pool is recruited near the viral factories but likely not into the VFs. It is clear that much remains unknown regarding the function and expression of RNPs and the interaction between vesicles and viral factories. However, the focus of this article is the identification of the interaction between the C-terminus of the polymerase and active Rab11a, and it provides us with solid evidence on this point.

Minor issues

1. For clarity, lines 146–147 state: "To identify the viral protein that mediates vRNP-Rab11a binding, we performed co-IP experiments using HEK293T cells transiently co-expressing HA-Rab11a and one of the vRNP proteins (N, P, L or M2-1) fused to GFP." However, Figure 1A does not include an L-GFP condition alone. Please revise the text to indicate that L was co-expressed with P. In addition, please move the subsequent sentence (lines 156–157), "Although GFP-L was co-expressed with P to ensure its stability, Rab11a specifically co-immunoprecipitated with the L-P complex but not with P alone, indicating that L mediates the interaction with Rab11a," immediately after the introductory description of the experimental design.

We applied these recommendations in the Results section *The interaction between vRNPs and Rab11a is mediated by the RSV Polymerase L* (p. 7-8) "we performed co-IP experiments using HEK-293T cells transiently co-expressing HA-Rab11a and one of the vRNPs proteins (N, P, L **co-expressed with P** or M2-1) fused to GFP. **[...] Although GFP-L was co-expressed with P to ensure its stability, HA-Rab11a specifically co-immunoprecipitated with the L-P complex but not with P alone, indicating that L mediates the interaction with Rab11a.** ~~Only the viral polymerase L enabled co-precipitation of HA-Rab11a when P, N, M2-1, and M failed to co-precipitate Rab11a.~~ **None of the other viral proteins tested (N, M2-1, or M) co-precipitated HA-Rab11a."**

2. The same issue applies to Figure 1C, where no L-GFP-alone condition is shown. Please revise the corresponding text accordingly.

We applied this recommendation in the Results section *The interaction between vRNPs and Rab11a is mediated by the RSV Polymerase L* (p. 8) "HEK-293T cells were transiently co-

transfected with one expression plasmid encoding either N, P, L **co-expressed with P**, M2-1 or M, alongside either an empty plasmid or a HA-Rab11a expression plasmid (Fig 1C)."

3. In Figure 1C, the Rab11a staining in the P-alone condition does not resemble the expected Rab11a localization and appears inconsistent with the other panels. Please verify that the correct image has been included.

This is the correct image. The limited resolution of the submitted format caused this impression. We have provided a higher-quality image below, and we will submit a higher-resolution image using the PLOS NAAS tool.

Submitted

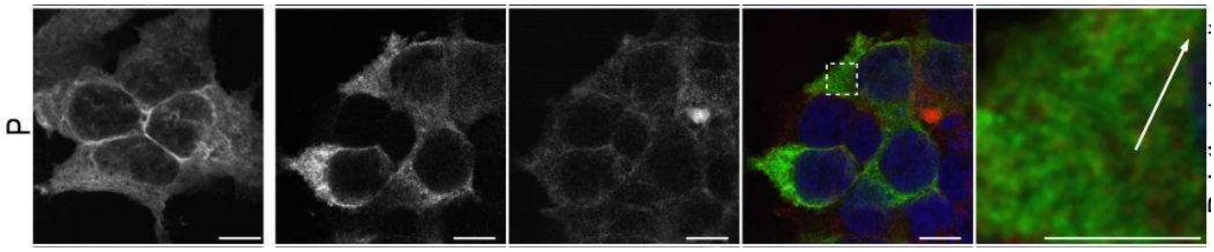

Better resolution image

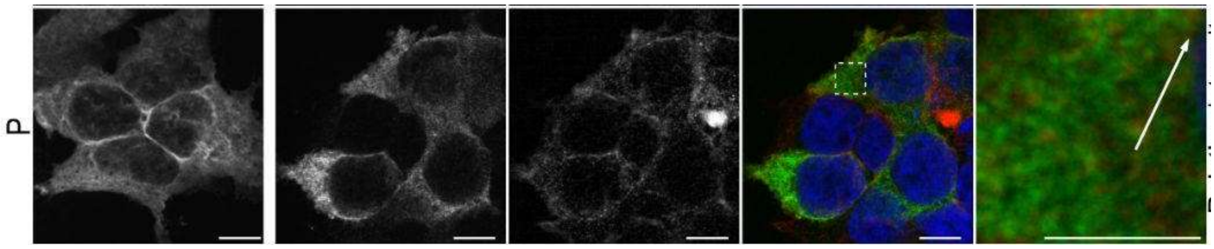

4. In Figure 3A, do the authors have an explanation for why the measured Kd is approximately four-fold lower (i.e., the affinity is higher) for HA-Rab11a WT? One would expect the affinities to be broadly similar or, if anything, for the constitutively active Rab11a mutant to exhibit the higher affinity. Such higher affinity of the Rab11CA construct is apparent in Figure 6E. In addition, could the authors include immunofluorescence images showing co-expression of the P(CTD) construct and L? This would be very useful ???

We agree that overall our datasets show that in cells Rab11aCA interacts better than Rab11a WT, CA being constitutively locked in its active conformation while WT can be in both active and inactive form. *In vitro* however, purified Rab11a WT with an excess of GTP in a test tube is by design more stable during the length of the experiment than in cells, which allows it to bind the L-P complex very well. Furthermore, the Kd measured by BLI is simply not precise enough to affirm that the affinity is lower for Rab11a WT: As we reported, the respective 95% confidence intervals are wide and overlapping : 0.1 to 1.2 μM for Rab11a WT, and 0.5 to 3 μM for Rab11a CA.

Lastly, the experimental setups of the Pull Down and BLI experiments are different and complimentary. They relied on distinct batches of protein complexes of L+PCTD and L+P full-length with a C-terminal his-tag, respectively, as also mentioned in the text and methods. Since they both confirm the specificity of the binding to GST-Rab11a WT+GTP and

Rab11aCA, together they reinforce the strengths of our findings. As we also have established at this stage that P itself is not necessary for the binding and the formation of the Rab11a-L aggregates (see co-immunoprecipitation experiment in Sup Fig 5 and the images below provided in our first response), we cannot justify going further in depth with colocalisations assays with truncations of P.

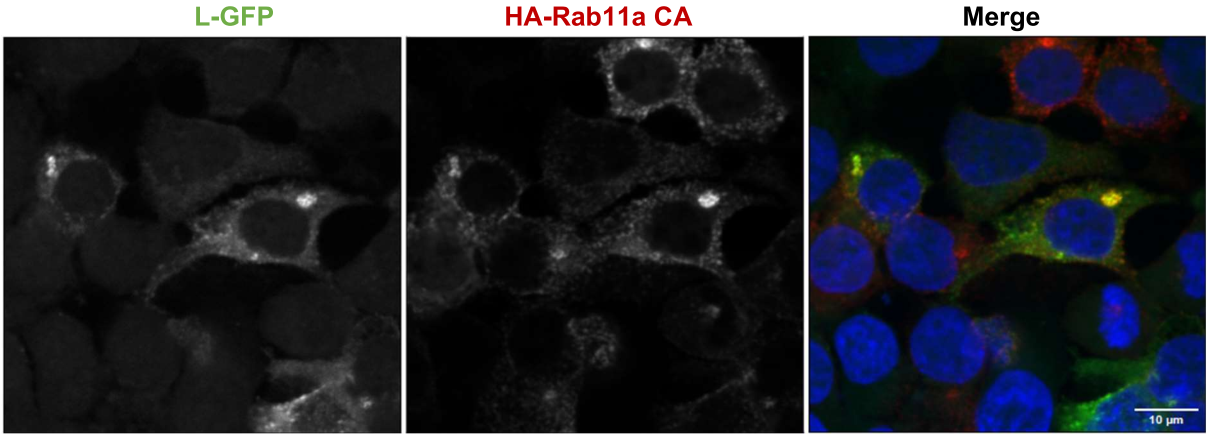

Fig legend: Polymerase L colocalizes with Rab11a. HEK 293T cells were transfected with constitutively active (CA, Q70L) HA-Rab11a together with L-GFP for 24h. Cells were fixed, immunolabelled and imaged using confocal microscopy to reveal L-GFP (green), HA-Rab11a (red) and nuclei (blue, Hoechst). Scale bars: 10 µm

5. Please correct the L-GFP+P panel in Figure 5D.

Figure 5D has been modified, as figures 1C, 2C, 7B and S4B for homogeneity.

6. In Figure 6B, the immunofluorescence images do not convincingly demonstrate co-localization between any of the L domains and Rab11a. Instead, the L fragments appear largely diffuse throughout the nucleus and cytoplasm without obvious enrichment at Rab11a-positive structures. a possibility is to change this panel to the supplementary material because it does not add information to the figure.

While we do find the colocalisation clear on our screen, we agree that the main information from this figure is carried by the immunoprecipitation, while the colocalisation only further supports this findings.

We therefore accepted the suggestion to move it in the supplementary section, which indeed makes the figure more concise and impactful.

8. In line 197, please capitalize "HA-Rab11a." The sentence should read: "The indicated viral protein was expressed alone (left column) or together with HA-Rab11a."

This has been corrected.
