# Supplementary material for: Direct interaction between RSV polymerase L and active Rab11a mediates viral ribonucleoprotein transport to assembly sites
Source: PLoS Pathog. 2026 Aug 3;22(8):e1013671. doi: 10.1371/journal.ppat.1013671 (PMC13456277; doi:10.1371/journal.ppat.1013671)
Supplement: Supplementary file 2 [file ppat.1013671.s014.docx]

PPATHOGENS-D-25-02679

Direct interaction between RSV polymerase L and active Rab11a mediates viral ribonucleoprotein transport to assembly sites

PLOS Pathogens

Dear Dr. Rameix-Welti,

Thank you for submitting your manuscript to PLOS Pathogens. After careful consideration, we feel that it has merit but does not fully meet PLOS Pathogens's publication criteria as it currently stands, but as will be clear from the reviewers comments has potential. Therefore, we invite you to submit a revised version of the manuscript that addresses the points raised during the review process.

We understand that the amount of work requested by the reviewers is substantial and given that we may not be able to commit to accept the revised version, we acknowledge that you may want to publish the manuscript in another journal. There are key points that the reviewers have asked that need attention, and in particular the ones listed below are critical:

Please note that the page numbers cited in this document refer to the revised manuscript with track changes.

**1) Rab11a function in RSV replication**

The importance of Rab11a in the RSV replication cycle is not directly examined.

We thank the reviewers for this insightful comment. We assessed the role of Rab11a in the RSV replication cycle by quantifying RSV multiplication over time in cells expressing wild-type (WT) or dominant-negative (DN) Rab11a, which were infected at a low multiplicity of infection (MOI). A549 cells stably expressing HA-Rab11a WT or DN were infected with RSV-GFP at an MOI of 0.005, and the GFP signal reflecting viral replication was measured every four hours for 72 hours (Rameix-Welti et al, 2014, doi: 10.1038/ncomms6104). We observed that cells expressing HA-Rab11a DN exhibited significantly reduced viral multiplication, with a ~50% decrease in GFP intensity at 72 hours post-infection compared to HA-Rab11a WT. These results suggest that Rab11a plays a role in RSV multiplication.

**Data are presented in Figure 8F and 8G**, and the following texts have been added :

Results section *The L-Rab11a interaction is required for efficient vRNPs transport in infected cells* (p. 36): “**To further evaluate the significance of this transport step, and of Rab11a in general, for RSV replication and spread, we examined viral replication kinetics in A549 cells stably expressing either HA-Rab11aWT or DN infected with RSV-GFP at a low multiplicity of infection. Viral multiplication was quantified by measuring the GFP signal every four hours over 72 hours post infection [31]. At each time point, GFP levels in cells expressing Rab11a DN were lower than in cells expressing Rab11a WT (Fig. 8F). At 72 hours post-infection, the GFP signal was ~50% lower in cells expressing HA-Rab11a DN than in cells expressing HA-Rab11a WT, indicating that the DN mutant impairs RSV replication (Fig. 8G). This confirms the involvement of Rab11a in RSV multiplication.**”

Fig 8 legend (p. 39): “**(F, G) Effect of Rab11a DN on viral growth. A549-HA-Rab11a-WT and A549-HA-Rab11a-DN cells were infected with RSV-GFP at an MOI of 0.005 and GFP fluorescence level was measured every 4h for 72h. (F) Scatter plot of GFP signal at different time post-infection in A549-HA-Rab11a-WT (green) or -DN (red). The data shown are the mean ± SD of four independent experiments performed in quadruplicate. (G) GFP intensity in infected A549-HA-Rab11a-DN cells at 72 hpi relative to WT. **** p* < 0.0001 by unpaired t-test with Welch's correction.**”

Discussion section “As Rab11a is involved **in RSV multiplication by mediating** **the transport of vRNPs** and has been shown to move with the vRNPs, it is tempting to speculate that the interaction between L and Rab11a **is responsible for the movement** of vRNPs.”

Materials and methods section :

- “Cells : **A549 cells constitutively expressing HA-Rab11a WT or HA-Rab11a DN are a kind gift from Dr. Nadia Naffakh [51] and were grown in DMEM supplemented with 1μg/mL puromycin**.” (p.45)
- “Virus : **The recombinant wild-type RSV, RSV-GFP** and RSV-GFP-N are derived from the human RSV Long strain sequence and were previously described [**31**,20].**”** (p.46)
- “**Viral multiplication assays :** **A549 cells stably expressing HA-Rab11a WT or DN were seeded in 24-well plates (1.25 × 10⁵ cells per well) in quadruplicate, 24 h prior to infection with RSV-GFP at a multiplicity of infection (MOI) of 0.005. GFP fluorescence was monitored every 4 h for 72 h post-infection using an IncuCyte S3 live-cell imaging system (Essen Bioscience), with images acquired using a 10× objective (36 fields per well). Fluorescence signals were analyzed using the IncuCyte software by quantifying the total green object integrated intensity (GCU × µm² per image) for each well at each time point. The percentage of integrated GFP intensity in HA-Rab11a DN-expressing cells relative to HA-Rab11a WT controls was calculated from the mean integrated GFP intensity across quadruplicate wells at 72 h post-infection. Statistical analyses and data visualization were performed using GraphPad Prism (GraphPad Software).”** (p.59)

2) Experimental and interpretative weaknesses

The manuscript is weakened by recurring issues such as missing controls, inconsistent quantification, unclear interpretation of aggregation phenotypes, and incomplete methodological descriptions.

These points were taken into account and addressed in the responses to the 3 reviewers

3) Long-range transport and downstream consequences

The authors conclude that vRNP long-range transport is impaired, yet they do not investigate effects on virus release or genome incorporation—processes that should also be impacted. Ideally, these experiments should be performed in Rab11a-knockout cells complemented with Rab11a-WT, CA, or DN to confirm Rab11a dependence. Moreover, if the minimal L fragment only partially competes with full-length L, one would expect some long-range trafficking events to persist; this possibility should be addressed.

We thank the reviewers for this important comment. Assessing the specific impact of impaired vRNP transport on downstream processes such as virus release or genome incorporation remains technically challenging in our system, although these processes are indeed expected to be affected. Notably, disruption of long-range transport is not complete, even upon Rab11a depletion by siRNA, as previously reported (Cosentino et al. 2022, PMID: 35797399), suggesting the existence of compensatory pathways. In addition, in our cellular models, RSV particles tend to remain associated with the cell surface, which makes accurate quantification of virus release difficult (Marougka et al. 2024; PMID: 39561185). Nevertheless, our data showing a significant reduction in viral replication kinetics in cells expressing Rab11a DN (see Response 1) support a functional role for Rab11a-dependent transport in the RSV replication cycle and viral spread. We agree that the use of Rab11a knockout cells complemented with Rab11a WT, CA, or DN would provide a powerful system to further establish Rab11a dependence. However, this approach is currently limited by the low efficiency of combined transfection and RSV infection.

Regarding the reviewer’s point on partial competition, we acknowledge that this aspect was not sufficiently clarified in the original manuscript. We have now addressed this point as follows:

Results section *The L-Rab11a interaction is required for efficient vRNPs transport in infected cells* (p. 36) : “The moderate effects observed may **be due to the** competitive nature of our inhibition approach**.** **In this approach, the endogenous full-length L protein can still compete with the L_1756-2165_ fragment for binding to Rab11a, which could enable residual vRNps transport.”**

Discussion section (p. 41): “**This would allow some long-range trafficking events to persist.**”

4) Crosslinking in pulldown assays

Crosslinker is applied throughout the pulldown assays, suggesting a transient or unstable interaction, which seems inconsistent with the BLI data showing stable binding. Including non-crosslinked pulldown assays would strengthen the manuscript by revealing whether the interaction is stable under native conditions.

We thank the reviewers for this insightful comment. Under native conditions (i.e., in the absence of crosslinking), we were able detect the L-Rab11a interaction by pulldown assays, but the signal-to-background ratio was low and the results were not consistently reproducible. These experiments did suggest the presence of a specific interaction, albeit weak and/or transient. In contrast, *in vitro* interaction is stable as demonstrated by pull down and biolayer interferometry (BLI) experiments. The transient nature of the interaction in a cellular context is possibly due to the high turnover of Rab11a between its active and inactive states in the presence of guanine nucleotide exchange factors (GEFs) and GTPase-activating proteins (GAPs).

To address this point, we have now included pulldown assays performed both in the absence and presence of crosslinker in **Figure S1A** and have clarified this point in the revised manuscript as follows:

Results section *The interaction between vRNPs and Rab11a is mediated by the RSV Polymerase L* (p. 7) : “**Noteworthy immunoprecipitation experiment performed under native conditions also revealed an association between L and Rab11a, but the signal was weak and inconsistent (example on Fig S1).**”

Supporting information (p.64) : “**S1 Fig. Co-immunoprecipitation analysis in the absence or presence of crosslinking. HEK-293T cells were transiently transfected with HA-Rab11a together with individual viral proteins (GFP-N, P-BFP, or L-GFP co-expressed with P) for 24 h. Cells were either left untreated (A) or treated with the crosslinking agent DSP for 30 min (B) prior to lysis. Cell lysates were subjected to immunoprecipitation using GFP-Trap beads to isolate tagged viral proteins. Input and bound proteins were analyzed by western blotting using anti-GFP, anti-P (for P-BFP and P detection), and anti-HA antibodies. * indicates P-BFP band. The lower band likely corresponds to a degradation product. Representative results from two independent experiments are shown.”**

5) Rab11a dominant-negative localization

Immunofluorescence data in RSV-infected cells are needed to show whether Rab11a-DN fails to colocalize with L, directly supporting the proposed mechanism. A comparison with Rab11a-WT or CA should also be provided.

We thank the reviewers for this important suggestion. To address this point, we performed immunofluorescence analyses in RSV-infected A549 cells stably expressing HA-Rab11a WT or DN. Cells were infected with RSV WT, and vRNPs were detected using an anti-N antibody, while HA-Rab11a was visualized using an anti-HA antibody. In cells expressing HA-Rab11a WT, Rab11a displayed a vesicular distribution and showed partial co-localization with vRNPs, consistent with the published data in Cosentino et al. 2022, PMID: 35797399. In contrast, HA-Rab11a DN exhibited a diffuse cytoplasmic localization and no longer formed discrete vesicular structures. Under these conditions, we did not observe the association between Rab11a DN and vRNPs. Because HA-Rab11a DN does not form discrete puncta, quantitative co-localization analysis could not be reliably performed. However, the clear change in Rab11a distribution and the loss of visible association with vRNPs support the conclusion that Rab11a vesicular organization is required for its interaction with vRNPs.

These results are now presented **in Figure S2B** and described in the revised manuscript as follows:

Results section *The polymerase L preferably interacts with the GTP-bound form of Rab11a* (p. 13) : “**To determine whether Rab11a-DN failed to colocalise with vRNPs in infected cells, we analysed the distribution of vRNPs relative to Rab11a-DN in RSV-infected cells. In RSV-infected A549 cells expressing HA-Rab11a WT (A549-Rab11aWT), Rab11a displayed a vesicular distribution and showed partial colocalisation with vRNPs, in line with previous observations [20]. In contrast, HA-Rab11a DN exhibited diffuse cytoplasmic localisation and no longer formed discrete vesicular structures. Under these conditions, no apparent association between Rab11a DN and vRNPs was observed (S2B Fig)**.”

Supporting information (p.65) “S2 Fig. (**B) Rab11a and vRNPs location in RSV infected cells. A549-HA-Rab11a WT or DN cells were infected with RSV (MOI 1) and fixed at 18hpi. Cells were immunolabelled and imaged using confocal microscopy to visualize vRNPs (anti-N antibody, green), HA-Rab11a (anti-HA antibody, red) and nuclei (Hoechst, blue). Scale bars: 10 μm (merge), 5 μm (zoom).** **Images are representative of two independent experiments.**”

6) Mapping the Rab11 binding site on L

A central question is whether Rab11 binds the MTase domain, the CTD, or a site spanning both domains (Figure 5). Several sentences imply that both domains are required (e.g., line 423), though it remains likely that only one domain contains the binding site. Repeating the pulldowns with GFP-L constructs expressing only the MTase domain or only the CTD would clarify which domain is necessary for the interaction.

We thank the reviewers for raising this important point. To investigate the Rab11a binding site on L, we performed co-immunoprecipitation assays using GFP-tagged fragments that corresponded to the C-terminal region (1756-2165) and the individual MTase (1756-2039) and CTD (2040-2165) domains. Our results show that neither the MTase nor the CTD domains resulted in the detectable co-precipitation of Rab11a, unlike the L_1756-2165_ fragment. This demonstrates that the integrity of the L_1756-2165_ fragment is necessary for effective binding. This suggests that either both regions contribute to the interaction, or subdividing these domains further disrupts their proper folding and structural integrity. Consequently, it is difficult to draw a solid conclusion on this matter.

These observations are now included **in the Fig S4A** and this point is addressed in the revised manuscript :

Results section *The two C-terminal domains of L are sufficient and essential for interaction with Rab11a* (p. 27) : “Consistent with our immunofluorescence results **the N-terminal fragment L_1-1755_-GFP failed to co-precipitate HA-Rab11a in any conformational state when the** full-length L (L_1-2165_) and the C-terminal fragment GFP-L_1756-2165_ specifically co-precipitated HA-Rab11a WT and CA but not HA-Rab11a DN. **We further subdivided the C-terminal fragment GFP-L_1756-2165_ into the MTase domain (L_1756-2039_) and the CTD (L_2040-2165_).** **Neither of these domains was sufficient to mediate co-precipitation of HA-Rab11a CA when expressed individually (S4A Fig). This suggests that either both regions contribute to the interaction, indicating that the binding site lies at the interface, or that subdividing these domains further disrupts their proper folding and structural integrity, thereby preventing any solid conclusion.”**

Discussion section (p. 43) : “**Similarly to our study, it was not possible to subdivide the C-terminal region of the influenza A virus PB2 subunit without disrupting any further its interaction with Rab11a [26]. While the C-terminal region of RSV L is typically divided into MTase and CTD domains based on sequence similarities with other viral polymerases, our findings indicate that these domains may not adopt a proper conformation when expressed individually and may require the integrity of the entire L_1756-2165_ region to form a functional Rab11a-binding interface**.”

Materials and methods section *Plasmids* (p. 47) : “**The pCI-GFP-L_1756-2039_ and pCI-GFP-L_2040-2165_ constructs were generated by PCR-mediated deletion from the pCI-GFP-L_1756-2165_ plasmid, followed by In-Fusion cloning (Takara Bio).**”

Supporting information (p.66) : **S4 Fig.** “(**A) Co-immunoprecipitation analysis of L C-terminal fragments. HEK 293T cells transiently expressing for 24h HA-Rab11a(Q70L) CA, HA-Rab11a(S25N) DN, together with P and L-GFP constructs corresponding to the full C-terminal fragment (L_1756-2165_), the MTase domain (L_1756-2039_), or the CTD (L_2040-2165_). Cells were treated with DSP for 30 min, lysed and incubated with GFP-Trap beads. Input lysates and precipitated fractions were analyzed by Western blot using anti-GFP, and anti-HA antibodies. The MTase activity of the GFP-L_1756-2039_ is inactivated by mutating two residues (R1820A and G1855S). Representative of three independent experiments**.”

Please submit your revised manuscript by Feb 08 2026 11:59PM. If you will need more time than this to complete your revisions, please reply to this message or contact the journal office at plospathogens@plos.org. When you're ready to submit your revision, log on to https://www.editorialmanager.com/ppathogens/ and select the 'Submissions Needing Revision' folder to locate your manuscript file.

Please include the following items when submitting your revised manuscript:

* A rebuttal letter that responds to each point raised by the editor and reviewer(s). You should upload this letter as a separate file labeled 'Response to Reviewers'. This file does not need to include responses to any formatting updates and technical items listed in the 'Journal Requirements' section below.

* A marked-up copy of your manuscript that highlights changes made to the original version. You should upload this as a separate file labeled 'Revised Manuscript with Track Changes'.

* An unmarked version of your revised paper without tracked changes. You should upload this as a separate file labeled 'Manuscript'.

If you would like to make changes to your financial disclosure, competing interests statement, or data availability statement, please make these updates within the submission form at the time of resubmission. Guidelines for resubmitting your figure files are available below the reviewer comments at the end of this letter.

We look forward to receiving your revised manuscript.

Kind regards,

Maria João Amorim, Ph.D

Academic Editor

PLOS Pathogens

Thomas Hoenen

Section Editor

PLOS Pathogens

Sumita Bhaduri-McIntosh

Editor-in-Chief

PLOS Pathogens

orcid.org/0000-0003-2946-9497

Michael Malim

Editor-in-Chief

PLOS Pathogens

orcid.org/0000-0002-7699-2064

Journal Requirements:

1) We do not publish any copyright or trademark symbols that usually accompany proprietary names, eg ©, ®, or TM (e.g. next to drug or reagent names). Therefore please remove all instances of trademark/copyright symbols throughout the text, including:

- TM on page: 35.

2) We note that your Data Availability Statement is currently as follows: "All relevant data are within the manuscript and its Supporting Information files.". Please confirm at this time whether or not your submission contains all raw data required to replicate the results of your study. Authors must share the “minimal data set” for their submission. PLOS defines the minimal data set to consist of the data required to replicate all study findings reported in the article, as well as related metadata and methods (https://journals.plos.org/plosone/s/data-availability#loc-minimal-data-set-definition).

For example, authors should submit the following data:

1) The values behind the means, standard deviations and other measures reported;

2) The values used to build graphs;

3) The points extracted from images for analysis..

Authors do not need to submit their entire data set if only a portion of the data was used in the reported study.

If your submission does not contain these data, please either upload them as Supporting Information files or deposit them to a stable, public repository and provide us with the relevant URLs, DOIs, or accession numbers. For a list of recommended repositories, please see https://journals.plos.org/plosone/s/recommended-repositories.

If there are ethical or legal restrictions on sharing a de-identified data set, please explain them in detail (e.g., data contain potentially sensitive information, data are owned by a third-party organization, etc.) and who has imposed them (e.g., an ethics committee). Please also provide contact information for a data access committee, ethics committee, or other institutional body to which data requests may be sent. If data are owned by a third party, please indicate how others may request data access.

Note: If the reviewer comments include a recommendation to cite specific previously published works, please review and evaluate these publications to determine whether they are relevant and should be cited. There is no requirement to cite these works unless the editor has indicated otherwise.

Reviewers' Comments:

Reviewer's Responses to Questions

Part I - Summary

Please use this section to discuss strengths/weaknesses of study, novelty/significance, general execution and scholarship.

Reviewer #1: This study by Giry et al. investigates the mechanistic role of Rab11a in the intracellular trafficking of respiratory syncytial virus (RSV) viral ribonucleoprotein complexes (vRNPs). The authors propose that the viral polymerase L protein directly binds GTP-loaded Rab11a and that this interaction drives Rab11a-dependent vRNP transport. By combining co-immunoprecipitation experiments, mutagenesis, biochemical assays, and imaging analyses, the authors map the interaction interface to the Rab11a Switch I region and the C-terminal domain of L. They further suggest that this interaction is necessary for efficient vRNP trafficking to the plasma membrane and therefore for virion assembly.

Overall, this manuscript highlights a potentially important and previously underexplored mechanistic link between RSV polymerase L and Rab11a, with implications for understanding vRNP trafficking. However, the importance of Rab11a in the replication cycle of RSV is not addressed directly. Furthermore, the manuscript is weakened by recurring issues with missing controls, inconsistent quantification, unclear interpretation of aggregation phenotypes, and incomplete

methodological descriptions. These concerns substantially limit the strength of the conclusions. The manuscript would also benefit from careful editorial revision to improve clarity and consistency.

Reviewer #2: General Assessment:

This manuscript by Giry et al., entitled “Direct interaction between RSV polymerase L and active Rab11a mediates viral ribonucleoprotein transport to assembly sites,” convincingly demonstrates that only GTP-bound Rab11a binds the RSV L polymerase. The study further shows that Rab11a binding does not affect polymerase activity and identifies a specific residue involved in the interaction. This work extends the findings of Cosentino et al., who demonstrated Rab11a interaction with vRNPs, and represents a well-executed and important contribution to the fields of virology and membrane trafficking.

Rab11a is a central factor in nucleocapsid trafficking across multiple RNA viruses, giving the work broad relevance. However, some conclusions rely on experiments performed only in duplicate rather than triplicate, and several conceptual gaps remain that should be addressed.

Reviewer #3: This work from Giry and colleagues describes in molecular detail the interaction between the host protein Rab11 and the RSV polymerase (L). This is a substantial body of work addressing a question of high importance in the RSV field, with significant implications for (the growing number) of other RNA viruses that also use the Rab11 pathway to traffic their genomes. The work uses a suite of orthogonal techniques to map the Rab11-RSV interaction on both the host and viral side, and extends what is known about this interaction beyond what is known for any Rab11-viral polymerase pair to date. The implications of these findings (particularly the identification of a putative Rab11 binding motif within L) are highly significant, and may lead to advances in antiviral development and further mechanistic insights for a range of Rab11-dependent RNA viruses. While the work generally employs a range of experimental strategies to prove orthogonal evidence for each conclusion (confocal/IP for instance), some discrepancies in controls need addressing (ie, equal binding of bait proteins, protein stability). The work described here has the potential to significantly advance the field, in particular if the conclusions regarding the relevant domain and point mutant in L that dictate Rab11 binding can be strengthened.

Part II – Major Issues: Key Experiments Required for Acceptance

Please use this section to detail the key new experiments or modifications of existing experiments that should be absolutely required to validate study conclusions.

Generally, there should be no more than 3 such required experiments or major modifications for a "Major Revision" recommendation. If more than 3 experiments are necessary to validate the study conclusions, then you are encouraged to recommend "Reject".

Reviewer #1: 1. The study would be substantially strengthened by directly assessing RSV replication in Rab11a knockout cells, available from several laboratories. Alternatively, Rab11a silencing could be used. Testing the Rab11a–L association in Rab11a knockout cells, particularly for imaging, pulldown, and polymerase assays, would also help avoid confounding effects from endogenous Rab11a.

We thank the reviewer for this insightful comment. We assessed the role of Rab11a in the RSV replication cycle by quantifying RSV multiplication over time in cells expressing wild-type (WT) or dominant-negative (DN) Rab11a, which were infected at a low multiplicity of infection (MOI) **(please see response to Editor, point 1 below).**

We assessed the role of Rab11a in the RSV replication cycle by quantifying RSV multiplication over time in cells expressing wild-type (WT) or dominant-negative (DN) Rab11a, which were infected at a low multiplicity of infection (MOI). A549 cells stably expressing HA-Rab11a WT or DN were infected with RSV-GFP at an MOI of 0.005, and the GFP signal reflecting viral replication was measured every four hours for 72 hours (Rameix-Welti et al, 2014, doi: 10.1038/ncomms6104). We observed that cells expressing HA-Rab11a DN exhibited significantly reduced viral multiplication, with a ~50% decrease in GFP intensity at 72 hours post-infection compared to HA-Rab11a WT. These results suggest that Rab11a plays a role in RSV multiplication.

**Data are presented in Figure 8F and 8G**, and the following texts have been added :

Results section *The L-Rab11a interaction is required for efficient vRNPs transport in infected cells* (p. 36): “**To further evaluate the significance of this transport step, and of Rab11a in general, for RSV replication and spread, we examined viral replication kinetics in A549 cells stably expressing either HA-Rab11aWT or DN infected with RSV-GFP at a low multiplicity of infection. Viral multiplication was quantified by measuring the GFP signal every four hours over 72 hours post infection [31]. At each time point, GFP levels in cells expressing Rab11a DN were lower than in cells expressing Rab11a WT (Fig. 8F). At 72 hours post-infection, the GFP signal was ~50% lower in cells expressing HA-Rab11a DN than in cells expressing HA-Rab11a WT, indicating that the DN mutant impairs RSV replication (Fig. 8G). This confirms the involvement of Rab11a in RSV multiplication.**”

Fig 8 legend (p. 39): “**(F, G) Effect of Rab11a DN on viral growth. A549-HA-Rab11a-WT and A549-HA-Rab11a-DN cells were infected with RSV-GFP at an MOI of 0.005 and GFP fluorescence level was measured every 4h for 72h. (F) Scatter plot of GFP signal at different time post-infection in A549-HA-Rab11a-WT (green) or -DN (red). The data shown are the mean ± SD of four independent experiments performed in quadruplicate. (G) GFP intensity in infected A549-HA-Rab11a-DN cells at 72 hpi relative to WT. **** p* < 0.0001 by unpaired t-test with Welch's correction.**”

Discussion section “As Rab11a is involved **in RSV multiplication by mediating** **the transport of vRNPs** and has been shown to move with the vRNPs, it is tempting to speculate that the interaction between L and Rab11a **is responsible for the movement** of vRNPs.”

Materials and methods section :

- “Cells : **A549 cells constitutively expressing HA-Rab11a WT or HA-Rab11a DN are a kind gift from Dr. Nadia Naffakh [51] and were grown in DMEM supplemented with 1μg/mL puromycin**.” (p.45)
- “Virus : **The recombinant wild-type RSV, RSV-GFP** and RSV-GFP-N are derived from the human RSV Long strain sequence and were previously described [**31**,20].**”** (p.46)
- “**Viral multiplication assays :** **A549 cells stably expressing HA-Rab11a WT or DN were seeded in 24-well plates (1.25 × 10⁵ cells per well) in quadruplicate, 24 h prior to infection with RSV-GFP at a multiplicity of infection (MOI) of 0.005. GFP fluorescence was monitored every 4 h for 72 h post-infection using an IncuCyte S3 live-cell imaging system (Essen Bioscience), with images acquired using a 10× objective (36 fields per well). Fluorescence signals were analyzed using the IncuCyte software by quantifying the total green object integrated intensity (GCU × µm² per image) for each well at each time point. The percentage of integrated GFP intensity in HA-Rab11a DN-expressing cells relative to HA-Rab11a WT controls was calculated from the mean integrated GFP intensity across quadruplicate wells at 72 h post-infection. Statistical analyses and data visualization were performed using GraphPad Prism (GraphPad Software).”** (p.59)

While Rab11a knockout systems would provide a complementary approach, the presence of endogenous Rab11a does not preclude interpretation of our results. The Rab11a DN mutant (S25N) is well-characterized and acts in a dominant-negative manner by sequestering Rab11a effectors, thereby functionally inhibiting endogenous Rab11a-dependent trafficking pathways (Ullrich et al., 1996 (DOI : 10.1083/jcb.135.4.913) ; van de Graaf et al., 2006 (DOI : 10.1128/MCB.26.1.303-312.2006). In addition, all imaging and pulldown analyses specifically monitor the exogenously expressed Rab11a constructs via the N-terminal HA tag using anti-HA antibodies, allowing us to distinguish their localization and behavior independently of endogenous Rab11a.

2. Many conclusions derive from overexpression that generates aggregates acknowledged by the authors as artifacts that do not occur during RSV infection. Given that availability of RSV expressing GFP-tagged L protein, it is surprising that endogenous Rab11a pulldown from infected cells was not attempted. Such data would greatly strengthen the manuscript. In addition, although the authors initially state that P is required for L stability, later pulldown assays (Fig. 5B, 5E, S5B) are performed without P. The rationale for these differing conditions should be explained.

We thank the reviewer for this important comment. The interaction between Rab11a and RSV vRNPs in infected cells has indeed been previously reported using recombinant RSV expressing GFP-tagged L (Cosentino et al., 2022, PMID: 35797399). As shown in that study (figure shown below), pulldown of GFP-L from infected cells leads to co-precipitation of the entire vRNP complex, including N and P. While this confirms the association of Rab11a with vRNPs, it does not allow identification of the specific viral protein mediating this interaction. Because Western blot detection of endogenous Rab11 is difficult, this was only done in cells stably expressing a Rab11a fused to HA tag for detection. For this reason, we performed co-immunoprecipitation and immunofluorescence experiments under overexpression conditions, which enabled us to analyze viral proteins individually and identify L as the Rab11a-interacting partner.

Regarding the role of P, we agree that clarification was needed. P is required for optimal stability and solubility of L and is therefore included in all *in vitro* experiments. To maintain consistency, we also included P in most cell-based assays. However, our previous work showed that, although less soluble, L expressed in the absence of P remains properly folded and competent for protein–protein interactions (Sourimant et al., 2015). This allowed us to express L without P in Fig. S4B (previously S5B) to specifically assess whether P is required for the L-Rab11a interaction.

We have clarified these points in the revised Results section *The two C-terminal domains of L are sufficient and essential for interaction with Rab11a* (p. 28) : “**Based on our previous work showing that L expressed in the absence of P, although less soluble, remains properly folded and capable of interacting with its partners [29], we tested whether P is required for the L-Rab11a interaction. Similar GST pull-down results were obtained with full-length L-GFP expressed without P (Fig S4C),** further confirming that P is dispensable for the L-Rab11a interaction.”

3. Fig. 7. The authors conclude that vRNP long-range transport is impaired, yet they do not examine effects on virus release or genome incorporation, which should also be impacted. Ideally, these experiments should be repeated in Rab11a-knockout cells complemented with Rab11a-WT, CA, or DN to confirm Rab11a dependence. Furthermore, if the minimal L fragment only partially competes with full-length L, some long-range trafficking events should remain; this possibility should be addressed.

We thank the reviewers for this important comment. Assessing the specific impact of impaired vRNP transport on downstream processes such as virus release or genome incorporation remains technically challenging in our system, although these processes are indeed expected to be affected. Notably, disruption of long-range transport is not complete, even upon Rab11a depletion by siRNA, as previously reported (Cosentino et al. 2022, PMID: 35797399), suggesting the existence of compensatory pathways. In addition, in our cellular models, RSV particles tend to remain associated with the cell surface, which makes accurate quantification of virus release difficult (Marougka et al. 2024; PMID: 39561185). Nevertheless, our data showing a significant reduction in viral replication kinetics in cells expressing Rab11a DN (see Response 1) support a functional role for Rab11a-dependent transport in the RSV replication cycle and viral spread. We agree that the use of Rab11a knockout cells complemented with Rab11a WT, CA, or DN would provide a powerful system to further establish Rab11a dependence. However, this approach is currently limited by the low efficiency of combined transfection and RSV infection.

Regarding the reviewer’s point on partial competition, we acknowledge that this aspect was not sufficiently clarified in the original manuscript. We have now addressed this point as follows:

Results section *The L-Rab11a interaction is required for efficient vRNPs transport in infected cells* (p. 36) : “The moderate effects observed may **be due to the** competitive nature of our inhibition approach**. In this approach, the endogenous full-length L protein can still compete with the L_1756-2165_ fragment for binding to Rab11a, which could enable residual vRNps transport.”**

Discussion section (p. 41): “**This would allow some long-range trafficking events to persist.**”

4. Loading controls (such as actin or GAPDH) and size markers are absent from multiple figures (e.g., 1A, 2A, 4B, 5E, 6A). Imaging experiments also lack untransfected cell controls for background staining.

To clarify this point, in our co-immunoprecipitation experiments, HA-Rab11a serves as an internal control in the input fractions, allowing us to verify comparable expression levels and similar transfection efficiency across samples. In addition, identical amounts of input lysates were incubated with GFP-Trap beads to ensure consistency between conditions. We have added molecular weight markers, to the revised figures where appropriate (Fig 1A, 6E, 6G and S4C).

Regarding the imaging experiments, untransfected cells are present within the fields shown and provide an internal reference for background staining. To further illustrate this point, we provide additional representative images highlighting untransfected cells below for clarity.

**Fig legend: control immunofluorescence centered on non-transfected cells.** HEK 293T cells transiently expressing proteins of interest for 24h were fixed, immunolabelled and imaged using confocal microscopy to reveal L-GFP (green), HA-Rab11a (red) and nuclei (blue, Hoechst). Scale bars: 10 μm

5. Reporting of replicates is inconsistent. Several quantifications include only two biological replicates despite figure legends stating that three were performed (e.g., Fig. 1D, S4B). For pulldown quantification, intensities should be normalized to the amount of L retrieved before comparisons across conditions. This issue is particularly evident in Fig. 2A. Without proper normalization, quantification may be misleading.

We thank the reviewer for this comment and acknowledge the inconsistencies. To clarify this, we have only mentioned the experiments used to perform the quantitative analysis for Figs. 1C, 5D, and 6B. For figures 1C and 6B, which correspond to screening-type experiments, we have now restricted the analysis to two representative experiments. For Figure 5D, all three biological replicates were quantified. The corresponding figure legends have been updated accordingly to accurately reflect the number of experiments analyzed.

Regarding pulldown quantification, we agree that careful normalization is important. However, Western blot quantifications are very imprecise due to the non-linearity of the signal. The quantifications shown are primarily intended to demonstrate reproducibility across independent experiments rather than to provide strict quantitative comparisons. Nevertheless, where appropriate (e.g., Fig. 2A and Fig. 5B, previously Fig. 4A), signal intensities were normalized to the amount of L recovered in the pulldown, and we removed the comment on the comparison of the rab11a WT and rab11a-CA capture and adjusted the conclusion regarding the loss of capture of mutated rab11a.

Result section *The polymerase L preferably interacts with the GTP-bound form of Rab11a* (p.12) :

~~“showed that Rab11a CA recovery was approximately twice that of Rab11a WT (Fig 2B) suggesting that the co-precipitated Rab11a WT represents the fraction of Rab11a in the active conformation.”~~

Legend Fig 2B (p.15): “HA-Rab11a band intensities were quantified using ImageLab software, normalized **to the amount of L-GFP bound to the beads, and then expressed relative** to HA-Rab11a CA co-precipitated with L-GFP (ratio = 1).”

Result section *The Switch I region of Rab11a is required for interaction with L* (p. 23) : “Co-immunoprecipitation assays revealed a complete **reduction** ~~loss~~ of interaction between L-GFP and HA-Rab11aCA_AAA_ under cross-linking conditions”

Legend Fig 5C (p. 26) : “Quantification of co-immunoprecipitation. HA-Rab11a band intensities were quantified using ImageLab software, normalized **to the amount of L-GFP bound to the beads, and then expressed relative** to unmutated (IGV) HA-Rab11aCA (ratio = 1). Bar graph shows mean ± SD from three independent experiments.”

6. Crosslinker is applied throughout the pulldown assays, suggesting that the interaction may be transient or unstable. This seems inconsistent with the BLI data showing stable binding. The manuscript would be strengthened by including non-crosslinked pulldown assays to assess native interaction stability.

We thank the reviewer for this insightful comment. Under native conditions (i.e., in the absence of crosslinking), we were able detect the L-Rab11a interaction by pulldown assays, but the signal-to-background ratio was low and the results were not consistently reproducible. These experiments did suggest the presence of a specific interaction, albeit weak and/or transient. In contrast, *in vitro* interaction is stable as demonstrated by pull down and biolayer interferometry (BLI) experiments. The transient nature of the interaction in a cellular context is possibly due to the high turnover of Rab11a between its active and inactive states in the presence of guanine nucleotide exchange factors (GEFs) and GTPase-activating proteins (GAPs).

To address this point, we have now included pulldown assays performed both in the absence and presence of crosslinker in **Figure S1A** and have clarified this point in the revised manuscript as follows:

Results section *The interaction between vRNPs and Rab11a is mediated by the RSV Polymerase L* (p. 7) : “**Noteworthy immunoprecipitation experiment performed under native conditions also revealed an association between L and Rab11a, but the signal was weak and inconsistent (example on Fig S1).**”

Supporting information (p.64) : “**S1 Fig. Co-immunoprecipitation analysis in the absence or presence of crosslinking. HEK-293T cells were transiently transfected with HA-Rab11a together with individual viral proteins (GFP-N, P-BFP, or L-GFP co-expressed with P) for 24 h. Cells were either left untreated (A) or treated with the crosslinking agent DSP for 30 min (B) prior to lysis. Cell lysates were subjected to immunoprecipitation using GFP-Trap beads to isolate tagged viral proteins. Input and bound proteins were analyzed by western blotting using anti-GFP, anti-P (for P-BFP and P detection), and anti-HA antibodies. * indicates P-BFP band. The lower band likely corresponds to a degradation product. Representative results from two independent experiments are shown.”**

7. It is unclear in which experiments GTP was added to activate Rab11a constructs. Even Rab11a-CA requires GTP loading, and conditions lacking supplemental GTP, such as Fig. 5G, make comparisons to prior assays difficult. A clear and consistent description of GTP usage is needed in both Methods and figure legends.

We thank the reviewer for this comment and agree that clarification was needed.

Rab11a and its mutants can bind endogeneous nucleotides in cells prior to purification, however they differ in their binding kinetics with GTP and GDP, and hydrolysis rates. For instance, another Rab11a-CA variant has been crystalised with endogeneous GTP (PDB: 5EZ5), so while our Rab11a-CA variants could have dissociated or hydrolysed a small amount of GTP, it was consistently active in our assays. Furthermore, exogenous GTP was specifically added to further confirm the GTP-sensitivity of *in vitro* experiments, namely the pulldown assays using purified proteins and the BLI experiments, where Rab11a WT was supplemented with 1 mM GTP to promote its GTP-bound state.

8. Fig. S6B shows that the L1860 mutation impairs polymerase activity, yet the manuscript claims retained MTase activity without direct evidence (lines 467-469). MTase assays should be included. Expression levels of L1860 relative to WT should also be shown.

We thank the reviewer for this important comment. The minigenome assay used in Fig. S5B reflects the overall activity of the viral polymerase complex, including RNA synthesis and mRNA capping/methylation steps. The residual activity observed (~25% of WT) indicates that the L1860A mutant retains partial polymerase function; however, this assay does not allow us to specifically assess MTase activity. Direct measurement of MTase activity would require purification of the mutant L protein and dedicated biochemical assays, which are technically demanding and beyond the scope of the present study.

Regarding protein expression, our co-immunoprecipitation data indicate that L WT and L_L1860A_ are expressed at comparable levels, suggesting that the observed functional differences are not due to major differences in protein abundance.

This point has been clarified in the result section *Residue L1860 of polymerase L is essential for Rab11a interaction* (p. 33) : “**This low however conserved activity in the minireplicon assay indicates** that the **MTase domain of the** L_L1860A_ is still enzymatically active.”

Reviewer #2: Major Comments:

1. Rab11a membrane association versus membrane-less RSV inclusions (discussion)

RSV inclusion bodies containing nucleocapsid and L are membrane-less organelles. Functional Rab11 binding typically requires membrane association because the properly folded Switch I domain is stabilized on membranes. How do the authors reconcile L interaction with Rab11a under these conditions? Are nucleocapsids or inclusion bodies interacting directly or indirectly with membrane-bound Rab11a? Please expand the discussion with relevant cell biology and membrane trafficking mechanisms to propose possible models.

We thank the reviewer for this insightful comment, which raises an important mechanistic question. We agree that RSV inclusion bodies are membrane-less organelles and that Rab11a function is typically associated with membrane-bound compartments, where its active (GTP-bound) form is stabilized. This raises the question of how membrane bound Rab11a can interact with vRNPs that are initially localized within inclusion bodies. We did not yet solve this apparent paradox. At present, the precise timing of the interaction between Rab11a and vRNPs remain unclear. In particular, it is not known whether Rab11a contributes to the export of vRNPs from inclusion bodies or acts at a later stage.

Based on our data, which demonstrate a preferential interaction between L and the active form of Rab11a, we favor a model in which vRNPs are first released from inclusion bodies through a yet undefined mechanism. Once in the cytoplasm, vRNPs could then be captured by membrane-associated Rab11a via a direct interaction with L, allowing their subsequent transport along recycling endosomes.

We have expanded the Discussion to include these considerations and to propose this working model (p. 41) : “One model is that newly synthesized vRNPs interact with Rab11a in its active GTP-bound form (and therefore most likely associated with membranes) **via** the L domain**. As VFs are membraneless organelles, this suggests that either some membrane compartments can “enter “ the VFs , or vRNPs are exported from the VF by an unknown mechanism, allowing them to be captured by Rab11a vesicles at the VF periphery before subsequent transport** along the microtubule network**. The latter scenario is supported by reports of the presence of membranes close to RSV VFs [40,41] or other Mononegavirales [42,43].”**

2. Rab11a dominant-negative localization (experiment)

The authors should provide immunofluorescence data in RSV-infected cells showing whether Rab11aDN fails to colocalize with L, which would directly support their mechanistic claims. Provide a comparison with Rab11a WT or CA.

We thank the reviewer for this important suggestion. To address this point, we performed immunofluorescence analyses in RSV-infected A549 cells stably expressing HA-Rab11a WT or DN. Cells were infected with RSV WT, and vRNPs were detected using an anti-N antibody, while HA-Rab11a was visualized using an anti-HA antibody. In cells expressing HA-Rab11a WT, Rab11a displayed a vesicular distribution and showed partial co-localization with vRNPs, consistent with the published data in Cosentino et al. 2022, PMID: 35797399. In contrast, HA-Rab11a DN exhibited a diffuse cytoplasmic localization and no longer formed discrete vesicular structures. Under these conditions, we did not observe the association between Rab11a DN and vRNPs. Because HA-Rab11a DN does not form discrete puncta, quantitative co-localization analysis could not be reliably performed. However, the clear change in Rab11a distribution and the loss of visible association with vRNPs support the conclusion that Rab11a vesicular organization is required for its interaction with vRNPs.

These results are now presented **in Figure S2B** and described in the revised manuscript as follows:

Results section *The polymerase L preferably interacts with the GTP-bound form of Rab11a* (p. 13) : “**To determine whether Rab11a-DN failed to colocalise with vRNPs in infected cells, we analysed the distribution of vRNPs relative to Rab11a-DN in RSV-infected cells. In RSV-infected A549 cells expressing HA-Rab11a WT (A549-Rab11aWT), Rab11a displayed a vesicular distribution and showed partial colocalisation with vRNPs, in line with previous observations [20]. In contrast, HA-Rab11a DN exhibited diffuse cytoplasmic localisation and no longer formed discrete vesicular structures. Under these conditions, no apparent association between Rab11a DN and vRNPs was observed (S2B Fig)**.”

Supporting information (p.65) “S2 Fig. (**B) Rab11a and vRNPs location in RSV infected cells. A549-HA-Rab11a WT or DN cells were infected with RSV (MOI 1) and fixed at 18hpi. Cells were immunolabelled and imaged using confocal microscopy to visualize vRNPs (anti-N antibody, green), HA-Rab11a (anti-HA antibody, red) and nuclei (Hoechst, blue). Scale bars: 10 μm (merge), 5 μm (zoom). Images are representative of two independent experiments.**”

3. Functional requirement of Rab11a for RSV replication (experiment)

Although the biochemical interaction is well supported, there remains no evidence that Rab11a function is required for productive RSV replication. Functional data would substantially strengthen the manuscript and provide definitive proof of biological relevance. The authors should provide growth kinetics of RSV in A549-Rab11a and A549-Rab11aDN cells and validate inhibition in cells expressing mCherry-L(1756–2165).

We thank the reviewer for this insightful comment. We assessed the role of Rab11a in the RSV replication cycle by quantifying RSV multiplication over time in cells expressing wild-type (WT) or dominant-negative (DN) Rab11a, which were infected at a low multiplicity of infection (MOI). A549 cells stably expressing HA-Rab11a WT or DN were infected with RSV-GFP at an MOI of 0.005, and the GFP signal reflecting viral replication was measured every four hours for 72 hours (Rameix-Welti et al, 2014, doi: 10.1038/ncomms6104). We observed that cells expressing HA-Rab11a DN exhibited significantly reduced viral multiplication, with a ~50% decrease in GFP intensity at 72 hours post-infection compared to HA-Rab11a WT. These results suggest that Rab11a plays a role in RSV multiplication.

**Data are presented in Figure 8F and 8G**, and the following texts have been added :

Results section *The L-Rab11a interaction is required for efficient vRNPs transport in infected cells* (p. 36): “**To further evaluate the significance of this transport step, and of Rab11a in general, for RSV replication and spread, we examined viral replication kinetics in A549 cells stably expressing either HA-Rab11aWT or DN infected with RSV-GFP at a low multiplicity of infection. Viral multiplication was quantified by measuring the GFP signal every four hours over 72 hours post infection [31]. At each time point, GFP levels in cells expressing Rab11a DN were lower than in cells expressing Rab11a WT (Fig. 8F). At 72 hours post-infection, the GFP signal was ~50% lower in cells expressing HA-Rab11a DN than in cells expressing HA-Rab11a WT, indicating that the DN mutant impairs RSV replication (Fig. 8G). This confirms the involvement of Rab11a in RSV multiplication.**”

Fig 8 legend (p. 39): “**(F, G) Effect of Rab11a DN on viral growth. A549-HA-Rab11a-WT and A549-HA-Rab11a-DN cells were infected with RSV-GFP at an MOI of 0.005 and GFP fluorescence level was measured every 4h for 72h. (F) Scatter plot of GFP signal at different time post-infection in A549-HA-Rab11a-WT (green) or -DN (red). The data shown are the mean ± SD of four independent experiments performed in quadruplicate. (G) GFP intensity in infected A549-HA-Rab11a-DN cells at 72 hpi relative to WT. **** p* < 0.0001 by unpaired t-test with Welch's correction.**”

Discussion section “As Rab11a is involved **in RSV multiplication by mediating** **the transport of vRNPs** and has been shown to move with the vRNPs, it is tempting to speculate that the interaction between L and Rab11a **is responsible for the movement** of vRNPs.”

Materials and methods section :

- “Cells : **A549 cells constitutively expressing HA-Rab11a WT or HA-Rab11a DN are a kind gift from Dr. Nadia Naffakh [51] and were grown in DMEM supplemented with 1μg/mL puromycin**.” (p.45)
- “Virus : **The recombinant wild-type RSV, RSV-GFP** and RSV-GFP-N are derived from the human RSV Long strain sequence and were previously described [**31**,20].**”** (p.46)
- “**Viral multiplication assays :** **A549 cells stably expressing HA-Rab11a WT or DN were seeded in 24-well plates (1.25 × 10⁵ cells per well) in quadruplicate, 24 h prior to infection with RSV-GFP at a multiplicity of infection (MOI) of 0.005. GFP fluorescence was monitored every 4 h for 72 h post-infection using an IncuCyte S3 live-cell imaging system (Essen Bioscience), with images acquired using a 10× objective (36 fields per well). Fluorescence signals were analyzed using the IncuCyte software by quantifying the total green object integrated intensity (GCU × µm² per image) for each well at each time point. The percentage of integrated GFP intensity in HA-Rab11a DN-expressing cells relative to HA-Rab11a WT controls was calculated from the mean integrated GFP intensity across quadruplicate wells at 72 h post-infection. Statistical analyses and data visualization were performed using GraphPad Prism (GraphPad Software).”** (p.59)

Performing RSV growth kinetics in cells expressing mCherry-L_1756-2165_ is technically challenging due to the low efficiency of combined transfection and RSV infection in these cells. Under these conditions, only a small fraction of cells would both express the construct and be productively infected, making it difficult to obtain reliable and interpretable replication kinetics. As a result, such measurements would not accurately reflect the impact of the L_1756-2165_ fragment on RSV replication. Instead, we assessed the effect of this construct on vRNP transport using live-cell imaging, which allows direct analysis at the single-cell level and avoids these limitations.

Finally, although technically challenging and possibly outside the scope of the current study, it would be valuable to determine whether a virus containing the L1860 mutation can be rescued (optional).

We agree that generating a virus carrying the L1860 mutation would be highly interesting and could provide valuable insights into its role in RSV replication. However, rescuing and characterizing such a virus is technically challenging and would require substantial time and resources, making it beyond the scope of the current study. We consider this a promising direction for future investigations.

Reviewer #3: • A key question is whether Rab11 is binding to the MTase domain, the CTD, or a site that spans both domains (Figure 5). As currently written, many sentences describing this interaction imply that both domains are required (see line 423 for example), though I believe it is still likely that the actual binding site is in one of the two regions. Repeating the pulldowns with GFP-L constructs that express only the MTase domain or only the CTD should clarify which of the domains is required for the interaction.

We thank the reviewers for raising this important point. To investigate the Rab11a binding site on L, we performed co-immunoprecipitation assays using GFP-tagged fragments that corresponded to the C-terminal region (1756-2165) and the individual MTase (1756-2039) and CTD (2040-2165) domains. Our results show that neither the MTase nor the CTD domains resulted in the detectable co-precipitation of Rab11a, unlike the L_1756-2165_ fragment. This demonstrates that the integrity of the L_1756-2165_ fragment is necessary for effective binding. This suggests that either both regions contribute to the interaction, or subdividing these domains further disrupts their proper folding and structural integrity. Consequently, it is difficult to draw a solid conclusion on this matter.

These observations are now included **in the Fig S4A** and this point is addressed in the revised manuscript :

Results section *The two C-terminal domains of L are sufficient and essential for interaction with Rab11a* (p. 27) : “Consistent with our immunofluorescence results **the N-terminal fragment L_1-1755_-GFP failed to co-precipitate HA-Rab11a in any conformational state when the** full-length L (L_1-2165_) and the C-terminal fragment GFP-L_1756-2165_ specifically co-precipitated HA-Rab11a WT and CA but not HA-Rab11a DN. **We further subdivided the C-terminal fragment GFP-L_1756-2165_ into the MTase domain (L_1756-2039_) and the CTD (L_2040-2165_).** **Neither of these domains was sufficient to mediate co-precipitation of HA-Rab11a CA when expressed individually (S4A Fig). This suggests that either both regions contribute to the interaction, indicating that the binding site lies at the interface, or that subdividing these domains further disrupts their proper folding and structural integrity, thereby preventing any solid conclusion.”**

Discussion section (p. 43) : “**Similarly to our study, it was not possible to subdivide the C-terminal region of the influenza A virus PB2 subunit without disrupting any further its interaction with Rab11a [26]. While the C-terminal region of RSV L is typically divided into MTase and CTD domains based on sequence similarities with other viral polymerases, our findings indicate that these domains may not adopt a proper conformation when expressed individually and may require the integrity of the entire L_1756-2165_ region to form a functional Rab11a-binding interface**.”

Materials and methods section *Plasmids* (p. 47) : “**The pCI-GFP-L_1756-2039_ and pCI-GFP-L_2040-2165_ constructs were generated by PCR-mediated deletion from the pCI-GFP-L_1756-2165_ plasmid, followed by In-Fusion cloning (Takara Bio).**”

Supporting information (p.66) : **S4 Fig.** “(**A) Co-immunoprecipitation analysis of L C-terminal fragments. HEK 293T cells transiently expressing for 24h HA-Rab11a(Q70L) CA, HA-Rab11a(S25N) DN, together with P and L-GFP constructs corresponding to the full C-terminal fragment (L_1756-2165_), the MTase domain (L_1756-2039_), or the CTD (L_2040-2165_). Cells were treated with DSP for 30 min, lysed and incubated with GFP-Trap beads. Input lysates and precipitated fractions were analyzed by Western blot using anti-GFP, and anti-HA antibodies. The MTase activity of the GFP-L_1756-2039_ is inactivated by mutating two residues (R1820A and G1855S). Representative of three independent experiments**.”

• Fig 3D would fit better in a stand-alone figure focused on polymerase function- this is a key conclusion of the work, and should be highlighted as such. This should include data from the relevant supplemental figure (S3) with appropriate biological replicates. In particular, multiple replicates of the methyl transferase assay seem key here, as from the single rep shown it appears that the MTase activity decreases in the presence of the Rab11 DN construct- the lack of statistical significance could simply be that the N is too small.

We followed the reviewer's excellent suggestion and brought the following modifications:

Figure 3 panel D was transferred to a new main figure (**revised Figure 4)** together with data previously presented supplementary figure 3 dedicated to the impact of Rab11a on RSV L protein activity.

The paragraph order in the Results section *“Rab11a does not ~~impact~~ abrogate RSV polymerase activity”* (p. 19) has been adapted to match the data order in the new figure.

The MTase activity assay has then been redone with appropriate biological replicates (N=3). The MTase activity indeed decreases slightly with increasing concentrations of all Rab11a constructs, which is now discussed in the text.

Result section “*Rab11a does not ~~impact~~ abrogate RSV polymerase activity”* (p. 20) : **“Increasing concentrations of purified Rab11a-CA did not statistically significantly affect MTase activity compared to control conditions (Fig. 4B). A mild, dose-dependent reduction in RSV L polymerase MTase activity was observed in the presence of purified Rab11a WT and DN, but this was likely not related to L binding, as only Rab11a-CA binds to L in these experimental conditions (Fig. 3).** ~~This assay showed no significant differences in RSV polymerase MTase activity in the presence of purified Rab11a WT, CA, or DN compared to the GST control (S3C Fig). The methylation efficiency remained consistent across all tested conditions, indicating~~ **Altogether this suggests** that Rab11a binding **to RSV L** does not ~~modulate~~ **abrogate** the cap-modifying function of L ***in vitro****.”*

The polymerase activity assay using RSV minireplicon system has also been redone with the correct number of biological replicates (N=3) in BSRT7/5 cells. This confirmed that Rab11a does not significantly impact the transcription and replication functions of the viral polymerase complex in this cellular context. These updated data are presented in the new figure 4.

**New figure 4**

Legend was modified accordingly.

**“Fig 4: Rab11a interaction does not abrogate RSV L activity. (A) 3’ primer-extension assay. RSV L-Twin-Strep-tag + P-his (0.2 µM) was incubated with a fluorescently-labeled RNA primer annealed to a 16nt RNA template. The RdRp activity leads to accumulation of fluorescent RNA primers extended with 1-12 nucleotides. The RdRp activity was assayed in presence of GST-Rab11a WT, CA or DN (7 µM). The activity in presence of PBS (vehicle) or GST was included as negative controls for inhibition, while an inhibitor (PC786, 1µM) of the RSV L RdRp activity was included as a positive control for inhibition. Fluorescent primers extended by the RSV polymerase were separated by Urea-PAGE and their fluorescence detected by a fluorescence imaging system. Top: Representative gel from 3 independent experiments; Bottom: Densitometry analysis of the intensity of fully +12nt extended primers (N=3). (B) *in vitro* methyltransferase (MTase) activity assay. The RSV L-Twin-Strep-tag + P^CTD^ Methyltransferase activity was assayed in the presence of GST-Rab11a WT, CA or DN in vitro using an established protocol [26]. As a control, a known inhibitor of RSV methyltransferase activity (Sinefungin) was included (N=3). (C) Polymerase activity assay in presence of Rab11a WT, CA or DN mutants. BSRT7/5 cells were transfected with plasmids encoding N, P, L, M2-1, and M/Luc subgenomic minireplicon together with control GFP or HA- Rab11a WT, HA-Rab11a(Q70L) CA, HA-Rab11a(S25N) DN. Polymerase activity was measured by luciferase luminescence normalized to β-galactosidase activity. Data represent individual data points and mean ± SD (N=3). “**

Finaly we corrected an error, that does not affect our conclusions, in **the labeling of the gel lanes in Fig3A: GST-Rab11a CA and GST-Rab11a+GTP were inverted**, as well as GST-Rab11a and GST-Rab11a DN. Since they behave similarly the error was missed during assembly and was noticed during preparation of a supplementary file containing all replicates from our original files.

• The sequence similarities between the Rab11 binding domain of FIP2 and the residues of L within the 1756-2165 portion are very intriguing. However, the fact that the L1860A mutation has such a severe effect on polymerase function complicates the interpretation of Figure 6. What if Rab11 binds only to L that is functional, and thus the loss of the Rab11-L interaction is due to the severe defect in polymerase function mediated by the L1860A mutation. Does mutation of either of the two residues immediately upstream also decrease polymerase function so dramatically? (if not, they could get around this conundrum). Would such a mutation in the MTase domain be expected to have such a dramatic effect on the minireplicon assay in any case? (vs the MT assay)- my worry is that this implies that the protein in inherently unstable.Would the MTase catalytic site mutations have a similar effect on Rab11 binding? (which could help distinguish the motif around 1860 from MTase activity?)

We thank the reviewer for these insightful comments. To address whether the loss of Rab11a binding observed with the L1860A mutant could be an indirect consequence of impaired polymerase function, we compared it to the MTase-inactive mutants (R1820A and G1855S from the GEGAG catalytic site) within the L_1756-2165_ fragment, which were already used in this study. These MTase mutants retain structural integrity and were designed to avoid cellular toxicity (Sutto-Ortiz et al. 2021, doi:10.1371/journal.ppat.1009562). Importantly, despite having only ~10% residual activity in the minigenome assay, the MTase mutants still efficiently interact and colocalize with HA-Rab11a WT and CA (L_1756-2165_ fragment, Fig 6). In contrast, the L1860A mutant exhibits ~25% minigenome activity but fails to interact or colocalize with Rab11a. These data indicates that the loss of Rab11a binding and colocalization is not simply a consequence of reduced polymerase activity, but is specifically related to the L1860 region.

To clarify this point, the transcription-replication activity of this MTase inactive mutant was added to the **S5B Fig,** and the manuscript has been updated accordingly :

Results section “*The two C-terminal domains of L are sufficient and essential for interaction with Rab11a”* (p. 27) “**It is noteworthy that the MTase activity of the GFP-L_1756-2165_ was inactivated by mutating two residues of the catalytic site (R1820A and G1855S) to avoid cellular toxicity, while maintaining structural integrity [29].**"

Figure 6 legend (p.31) : “**The MTase activity of the GFP-L_1756-2165_ is inactivated by mutating two residues (R1820A and G1855S)**”

Results section “*Residue L1860 of polymerase L is essential for Rab11a interaction”* (p. 33) : “The L_L1860A_ mutant retained approximately 25% of wild-type polymerase activity in minigenome reporter assays (S6B Fig) **when a L mutated in the MTase catalytic site (L_R1820A,G1855S_) showed only ~10% residual activity.** **This low however conserved activity in the minireplicon assay indicates that the** MTase domain **of the L_L1860A_** is still enzymatically active.”

Materials and methods section *Plasmids* (p. 47) : “**MTase-inactivating mutations (R1820A and G1855S) were introduced into pCI-GFP-L_1756-2165_ and pCI-GFP-L_1756-2039_ by site-directed mutagenesis** ~~The L1860A point mutation was introduced into pCI-L-GFP by site-directed mutagenesis~~ using the In-Fusion HD Cloning Kit (Takara Bio)[29]. **The L1860A point mutation was introduced into pCI-L-GFP using the same approach.** Mutagenic primers were designed to **incorporate the desired substitutions** ~~replace leucine 1860 with alanine (L1860A)~~ and included 15 bp regions of homology flanking the mutation site...”

We did not mutate residues immediately upstream or downstream of L1860, as they are not predicted to participate in Rab11-FIP2 interaction with Rab11a.

Part III – Minor Issues: Editorial and Data Presentation Modifications

Please use this section for editorial suggestions as well as relatively minor modifications of existing data that would enhance clarity.

Reviewer #1:

In the abstract, the phrase “its involvement” is ambiguous and should be clarified.

This has been rephrased to clarify (p. 3)

**“**Competitive inhibition of ~~Rab11a-L~~ **the** interaction **between Rab11a and L** using the minimal Rab11a-binding domain ~~reveals its involvement in vRNPs’ transport~~ ~~as it~~ significantly impairs vRNP dynamics during infection, **indicating that Rab11a-L binding is involved in the transport of vRNPs**.”

In the introduction, it is unclear whether “ubiquitously expressed” refers to Rab11a, Rab11b, or both.

This has been clarified (p. 5) : “(**both** ubiquitously expressed)”

Fig. 1A: BFP-P shows multiple bands; please address.

The lower bands are likely degradation products, we added an asterisk and described it in the S1 fig legend (p. 64) : “*** indicates P-BFP band. The lower band likely corresponds to a degradation product**”. We replaced the P-BFP construct with a P-GFP construct to ensure optimum interaction with the beads and repeated the immunoprecipitation experiment (data are shown in Fig. 1A). The use of P-GFP resulted in increased capture of P-GFP and in a cleaner signal with reduced degradation.

Fig. 3: SEC profiles and purification gels for L+P should be shown.

We added a new supplementary figure with SEC profiles for L+P, as well as GST-Rab11a constructs, and an SDS-PAGE of peak fractions as a **supplementary Figure S3 panels A, B and C, and the appropriate legend.**

Fig. 3D: Clarify why two vehicle controls are included and explain the normalization strategy. Which bands were included in the quantification?

Two “vehicle” are present since we analyzed on the same gel the effect on RdRP activity of GST-Rab11a variants on one side and of other proteins unrelated to this project on the other side, since the gel broke on the top left in the first vehicle lane, we chose to keep the second vehicle lane for this figure presentation and for normalization. The 3 gels are shown below.

Additional experiments are also provided in the source file.

We updated the methods section *In vitro polymerase assay* (p. 57) to further describe the normalization strategy: “**After imaging, the intensity of the fully extended transcripts (+12nt) was measured using ImageJ software, and the signals were normalized to the vehicle control in each independent experiment (n=3).”**

Reviewer #2: Minor / Technical Comments:

1. Crosslinking and Western blot analysis

• Please explain the presence of three P-BFP bands.

The lower bands are likely degradation products, we added an asterisk and described it in the S1 fig legend (p. 64) : “* indicates P-BFP band. The lower band likely corresponds to a degradation product”. We replaced the P-BFP construct with a P-GFP construct to ensure optimum interaction with the beads and repeated the experiment in Fig. 1A. The use of P-GFP resulted in a cleaner signal with reduced degradation.

• Does the anti-GFP antibody recognize GFP and BFP with equal affinity?

The anti-GFP antibody does not efficiently detect BFP, which is why P-BFP was visualized using an anti-P antibody (now only in S1 Fig). We now performed immunoprecipitation experiments with a P-GFP instead of P-BFP and show these new results in figure 1. The P-GFP is better retained on the beads and well recognized by the anti-GFP antibody, but still failed to capture Rab11a.

The figure legend have been adjusted accordingly. Supporting information (p. 64) : “Input and bound proteins were analyzed by western blotting using anti-GFP, anti-P **(for P-BFP and P detection)**, and anti-HA antibodies.”

• Include L-only controls for co-IP western blots and IF to confirm specificity. Since L stability depends on P, L alone should not bind.

We have previously shown that although the L protein expressed without P is less soluble, it is folded and able to interact with its partners (Sourimant et al., 2015, DOI : 10.1128/jvi.03619-14). L is expressed at lower levels but still efficiently interacts with rab11a as shown on S4C Fig . It also co-localizes with Rab11a, as shown in the figures below.

**L-GFP**	**HA-Rab 11a CA**	**Merge**

**Fig legend: Polymerase L colocalizes with Rab11a.** HEK 293T cells were transfected with constitutively active (CA, Q70L) HA-Rab11a together with L-GFP for 24h. Cells were fixed, immunolabelled and imaged using confocal microscopy to reveal L-GFP (green), HA-Rab11a (red) and nuclei (blue, Hoechst). Scale bars: 10 μm

• Figure 1 appears to show only duplicate experiments. Please provide results from at least three independent replicates.

We have now repeated the co-immunoprecipitation experiments using P-GFP instead of P-BFP and performed three independent biological replicates. The corresponding results are presented in Figs. 1A and 1B.

For Figs. 1C-E, quantifications were performed on 20 cells from two independent immunofluorescence experiments. As these experiments correspond to a screening approach and yielded highly reproducible and consistent phenotypes across independent repeats, we limited the quantification to two experiments.

2. L truncation mapping

Please provide evidence that the truncated L constructs used for interaction mapping are properly folded. Loss of binding could result from misfolding rather than loss of the Rab11-interaction interface, limiting interpretation of negative results.

Our truncation strategy is based on previously published work from our group and others showing that these fragments are active and independently folded, which is also commonly observed for other L proteins of the mononegavirales order. This is also why shorter fragments were not included in these assays, as they are likely not functional and not folded properly. We added a short sentence in the text (in results section “*The two C-terminal domains of L are sufficient and essential for interaction with Rab11a “,* p. 27)

to clarify this point, "**Based on previous work characterizing the activity and structure of RSV L domains [28,29], we (...).** “

Note however, as requested in this revision we further attempted to truncate the Rab11a binding fragment into two smaller fragments that did not bind at all, likely as suggested due to an improper folding.

3. Figure 5G (GST pulldown)

• Why does GFP-L(1756–2165) migrate as two bands?

The lower band is likely a degradation product, we added an asterisk and described it in the figure 6 (former figure 5) legend.

• Why is binding to Rab11a WT weak?

In absence of exogenous GTP the WT Rab11a exists as a mixture of active and inactive forms, which is why the binding appears weak. Likely, adding another condition with exogenous GTP likely would increase the binding, however we already did it previously and focused instead here on testing the truncated L products.

• Was GTP included in the assay?

GTP was not included here, as we already established that Rab11a-CA and Rab11a-DN behave as the active and inactive form of Rab11a.

4. L1860 mutation

Please clarify the rationale for selecting L1860, especially as this residue does not reside within the GxGxG motif initially implicated. Why were not all glycines mutated initially? Were additional residues tested? Is this motif surface-exposed in the L structure? Please provide a structural figure highlighting the mutation site.

We thank the reviewer for this important comment. We selected residue L1860 for mutagenesis because it appeared as the most relevant candidate based on both sequence conservation and functional analogy. Specifically, this residue is conserved across sequences and corresponds to a position directly involved in the interaction between Rab11-FIP2 and Rab11a (PDB : 2GZD, Jagoe et al., 2006, DOI : 10.1016/j.str.2006.06.010). For this reason, we prioritized this mutation and have clarified this rationale in the revised manuscript. We did not test additional residues.

Result section *Residue L1860 of polymerase L is essential for Rab11a interaction* (p. 32) “**Moreover, it is the only residue conserved between RSV L and Rab11-FIP2 that has been shown to be directly involved in Rab11-FIP2 interaction with Rab11a (highlighted in yellow in the sequence alignment, Fig. S6A)[31].**”

Supporting information, S6A Fig (p. 67) : “**Residues previously shown to be critical for Rab11FIP2-Rab11a interaction are highlighted in yellow (PDB : 2GZD)[31].**”

Regarding structural considerations, there is no published structure of this region for RSV-L. Available structural predictions of RSV L suggest that L1860 is not strongly surface-exposed and is located in a loop immediately downstream of the GEGAG motif (see figure below). However, the MTase and CTD regions are known to be flexible and dynamic, and it is therefore possible that conformational changes could transiently expose this residue for interaction.

Cartoon representation of the 3D structure of RSV L (1756-2165) fragment modeled using alphafold3. MTase(1756-2039) and CTD(2040-2165) domains were colored in orange and red, respectively. L1860 is shown in a green spheres representation, while G1753, G1755, and G1757 are shown in purple spheres representations. These highlighted residues do not appear to be directly surface-exposed in this model.

5. L inclusion formation

Does L form cytoplasmic inclusions in the absence of GFP tagging?

We thank the reviewer for this question. The formation of cytoplasmic aggregates is not dependent on GFP tagging but rather on the conformational state of Rab11a and its ability to interact with L. To address this point, we performed similar experiments using L fused to an HA tag instead of GFP. Under these conditions, we still observed the formation of cytoplasmic aggregates in the presence of Rab11a WT and CA. Representative images are provided below.

Fig legend: Aggregation of L-HA and Rab11a. HEK 293T cells were transfected with wild-type (WT) mCherry-Rab11a or GFP- Rab11a, constitutively active (CA, Q70L) or dominant negative (DN, S25N) GFP-Rab11a together with L-HA and P for 24h. Cells were fixed, immunolabelled and imaged using confocal microscopy to reveal L-HA (red), Cherry or GFP-Rab11a (green) and nuclei (blue, Hoechst). Scale bars: 10 μm (main), 5 μm (zoom).

Reviewer #3: • BFP doesn’t bind as well to GFP trap (both in theory, and in Fig 1A where the efficiency of P-BFP pulldown vs the GFP pulldowns is very low. This makes it hard to completely rule out Rab11 binding to P (or a joint P/L epitope).

We thank the reviewer for this important comment. We agree that BFP does not bind as efficiently to GFP-Trap beads, which could limit the interpretation of the initial experiment. To address this concern, we generated a P-GFP construct and repeated the co-immunoprecipitation experiments three independent times. Despite efficient pulldown of P-GFP, we did not observe increased co-precipitation of HA-Rab11a in the absence of L, indicating that P alone is not sufficient to mediate the interaction with Rab11a. The updated data are now included in Fig. 1A, and the corresponding text has been revised accordingly.

Result section *The interaction between vRNPs and Rab11a is mediated by the RSV Polymerase L* (p. 7) “we performed co-IP experiments using HEK-293T cells transiently co-expressing HA-Rab11a and one of the vRNPs proteins (N, P, L or M2-1) fused to GFP **~~or BFP~~**.”

Materials and methods section *Plasmids* (p. 46) : “**The pCI-P-GFP was generated by replacing the BFP tag in pCI-P-BFP. The vector was digested with MluI and Eco81I, and the GFP insert obtained by gene synthesis (Twist Bioscience) was ligated using T4 DNA ligase.”**

• In Figure 1C the area chosen for line intensity scans are not easy to compare. The L-GFP+P condition has two distinct red peaks, representing discrete Rab11 positive structures. While I appreciate that the addition of L-GFP+P relocalizes Rab11 into multiple cytoplasmic structures, it would be more helpful for comparison to do the line intensity scans over the perinuclear Rab11 compartment (or another place in the image where there is a red peak of similar size/separation from background signal).

We thank the reviewer for this helpful suggestion. To facilitate comparison between conditions, we have replaced the line intensity scan shown for the P + Rab11a condition. The updated analysis was performed in a perinuclear region, similar to that used for the other conditions. The revised line scans are now presented in *Fig. 1C.*

• While the data in figure 2B argue convincingly that the DN version of Rab11 neither binds to L or induces its relocalization upon co-transfection, it is hard to make conclusions about the CA construct (Fig 2B). The efficiency of pulldown for L-GFP appears ~50% more efficient in the condition where it was co-transfected with Rab11 CA (vs the transfection with WT)- based on the L-GFP ‘bound’ lanes. While there is less HA-Rab11a in the ‘WT’ condition, there is less GFP-polymerase there as well, making it possible that the increased binding for CA is just reflective of more bound bait.

We thank the reviewer for this insightful comment. We agree that differences in pulldown efficiency could confound the comparison between Rab11a WT and CA. Moreover, quantifying western blot signals is hazardous due to their poor linearity. We may have overstated the significance of these data. To address this, we have adjusted the quantification of the co-immunoprecipitation experiments by normalizing the amount of HA-Rab11a co-precipitated to the quantity of L-GFP bound to the beads. The revised quantification is now presented in Fig. 2B, and the corresponding text has been updated to reflect this adjustment.

Result section *The polymerase L preferably interacts with the GTP-bound form of Rab11a* (p.12) :

~~“showed that Rab11a CA recovery was approximately twice that of Rab11a WT (Fig 2B) suggesting that the co-precipitated Rab11a WT represents the fraction of Rab11a in the active conformation.”~~

Legend Fig 2B (p.15): “HA-Rab11a band intensities were quantified using ImageLab software, normalized **to the amount of L-GFP bound to the beads, and then expressed relative** to HA-Rab11a CA co-precipitated with L-GFP (ratio = 1).”

• As in Fig 2B, the amount of L-GFP that is pulled down in the IGV and AAA conditions do not appear equal, which complicates the interpretation of this experiment. There is significantly more L-GFP present in the ‘Bound IGV’ sample vs the ‘Bound AAA’ sample, despite equivalent inputs, which raises the possibility that the differences in HA-Rab11 binding to L are the result of variations in binding efficiency. Multiple biological replicates with equal binding are needed to sort this out. In addition, it is difficult to assess changes in colocalization (Fig 4C), it would help to display single channel images of the zoomed in areas. But from the lower mag images it appears that the L-GFP is still found in areas of Rab11 concentration with the AAA construct (see perinuclear structure in lower left cell, large aggregate in upper right cell that appear enriched in both channels). Also, the quantification in Figure S4 should be moved into the main figure.

We thank the reviewer for this comment. We agree that differences in L-GFP pulldown efficiency could complicate interpretation. To address this, we have adjusted the quantification of the co-immunoprecipitation experiments by normalizing the amount of HA-Rab11a co-precipitated to the quantity of L-GFP bound to the beads. The revised quantification is now presented in Fig. 4B (updated to Fig. 5B in the revised manuscript), and the corresponding text has been updated to reflect this adjustment.

Result section *The Switch I region of Rab11a is required for interaction with L* (p. 23) : “Co-immunoprecipitation assays revealed a complete **reduction** ~~loss~~ of interaction between L-GFP and HA-Rab11aCA_AAA_ under cross-linking conditions”

Legend Fig 5C (p. 26) : “Quantification of co-immunoprecipitation. HA-Rab11a band intensities were quantified using ImageLab software, normalized **to the amount of L-GFP bound to the beads, and then expressed relative** to unmutated (IGV) HA-Rab11aCA (ratio = 1). Bar graph shows mean ± SD from three independent experiments.”

To facilitate visualization of colocalization in Fig. 4C (now Fig. 5C), we have added line scan analyses (in particular the perinuclear structures of the lower left cell of the lower image) and updated the text accordingly.

Finally, the quantification previously shown in Figure S4 has been moved into the main figure to improve clarity and accessibility.

PLOS authors have the option to publish the peer review history of their article (what does this mean?). If published, this will include your full peer review and any attached files.

If you choose “no”, your identity will remain anonymous but your review may still be made public.

Do you want your identity to be public for this peer review?

For information about this choice, including consent withdrawal, please see our

Privacy Policy

.

Reviewer #1: No

Reviewer #2: No

Reviewer #3: No

[NOTE: If reviewer comments were submitted as an attachment file, they will be attached to this email and accessible via the submission site. Please log into your account, locate the manuscript record, and check for the action link "View Attachments". If this link does not appear, there are no attachment files.]

Figure resubmission:

While revising your submission, we strongly recommend that you use PLOS’s NAAS tool (https://ngplosjournals.pagemajik.ai/artanalysis) to test your figure files. NAAS can convert your figure files to the TIFF file type and meet basic requirements (such as print size, resolution), or provide you with a report on issues that do not meet our requirements and that NAAS cannot fix.

After uploading your figures to PLOS’s NAAS tool - https://ngplosjournals.pagemajik.ai/artanalysis, NAAS will process the files provided and display the results in the "Uploaded Files" section of the page as the processing is complete. If the uploaded figures meet our requirements (or NAAS is able to fix the files to meet our requirements), the figure will be marked as "fixed" above. If NAAS is unable to fix the files, a red "failed" label will appear above. When NAAS has confirmed that the figure files meet our requirements, please download the file via the download option, and include these NAAS processed figure files when submitting your revised manuscript.

Reproducibility:

To enhance the reproducibility of your results, we recommend that authors of applicable studies deposit laboratory protocols in protocols.io, where a protocol can be assigned its own identifier (DOI) such that it can be cited independently in the future. Additionally, PLOS ONE offers an option to publish peer-reviewed clinical study protocols. Read more information on sharing protocols at https://plos.org/protocols?utm_medium=editorial-email&utm_source=authorletters&utm_campaign=protocols

In compliance with data protection regulations, you may request that we remove your personal registration details at any time.

(Remove my information/details)

. Please contact the publication office if you have any questions.
