# Supplementary material for: Direct interaction between RSV polymerase L and active Rab11a mediates viral ribonucleoprotein transport to assembly sites
Source: PLoS Pathog. 2026 Aug 3;22(8):e1013671. doi: 10.1371/journal.ppat.1013671 (PMC13456277; doi:10.1371/journal.ppat.1013671)
Supplement: Supplementary file 4 [file ppat.1013671.s015.docx]

We would like to thank the reviewer for their comments. We are glad that our responses clarified the points and improved the manuscript. Our point-by-point responses to the remaining questions are provided below.

Please note that the page numbers cited in this document refer to the revised manuscript with track changes.

**Reviewers' Comments:**

 Reviewer's Responses to Questions

**Part I - Summary**
 Please use this section to discuss strengths/weaknesses of study, novelty/significance, general execution and scholarship.

Reviewer #1: In this revised version, the authors aim to further develop their mechanistic model in which RSV polymerase L directly interacts with GTP-bound Rab11a to mediate vRNP transport. The manuscript has improved in several aspects, including the addition of replication kinetics and further pulldown experiments. However, some of the concerns raised previously do not appear to be fully resolved and the new data are also somewhat problematic.
 Overall, the study addresses an important question and has clear potential. Nevertheless, in its current form, some issues remain that limit the strength of the conclusions and would benefit from additional clarification or experimental support.

Reviewer #2: The authors have addressed all my comments and provided the required experiements where ever it was possible. It is understandable that rescuing the L1860 virus and performing RSV growth kinetics in cells expressing mCherry-L1756-2165 is technically challenging and can be completed in future studies.

Reviewer #3: (No Response)

**Part II – Major Issues: Key Experiments Required for Acceptance**

Reviewer #1: 1. The statistical analysis remains problematic. The authors now report only two biological replicates, yet still obtain extremely low p-values (e.g., p < 0.0001) (Figure 1D, Figure 6C, D). This is not plausible given such low replicate numbers and suggests that individual cells may have been pooled and treated as independent observations. If so, this would constitute inappropriate statistical treatment and artificially inflate significance.

The quantification results are presented as follows: we report the accumulation of Rab11a in GFP-L aggregates, measured as Rab11a intensity within the aggregate relative to the surrounding cytoplasm, along with the percentage of cell surface occupied by aggregates.

Although the cells share common experimental conditions (day of seeding, transfection batch), they represent distinct biological entities that differ in transfection efficiency for each plasmid, cell cycle stage, and other individual parameters. They can therefore be analyzed as independent events. This is standard practice, as performing one experiment per cell is not feasible. Accordingly, the number of events reported corresponds to the total number of cells analyzed across two independent experiments. Representing only a single mean value per experiment would mask the true inter-cellular variability.

It should also be emphasized that these statistical analyses are not critical for the interpretation of the data. These quantitative measurements were performed to complement, rather than replace, representative images. While they have inherent limitations, the observed phenotype is unequivocal. Furthermore, all imaging results were systematically validated by co-immunoprecipitation and/or co-purification experiments confirming our quantification.

To report Rab11 concentration within aggregates, we initially displayed individual aggregates to illustrate the full dispersion of the data. We agree that averaging Rab11 concentrations across aggregates on a per-cell basis is more rigorous. The graphs in Figures 1E, 2D, 5F, 6D and S5E, the statistical analysis of the presented data and the corresponding legends have been revised accordingly.

Results section *The interaction between vRNPs and Rab11a is mediated by the RSV Polymerase L* (p. 8) : “the ratio of HA-Rab11a fluorescence intensity within aggregates to the median cellular HA-Rab11a intensity**, averaged per cell.”**

Fig 1 legend (p. 11) : “Dot plot shows mean ± SD, each point representing **the mean enrichment ratio of aggregates for one cell**~~one aggregate~~. *** *p* < 0.05; ** *p* < 0.01;** *** *p* < 0.~~0~~001~~,~~ **;** **** *p* < 0.0001 by ~~Brown-Forsythe and Welch~~**Kruskal-Wallis** ANOVA test followed by ~~Dunnett's~~ **Kruskal-Wallis** multiple comparisons test**s for L-GFP sample**. Data from n = 20 cells, two independent experiments. **No values are included for cells without aggregates.**”

Fig 2 legend (p. 16) : “Dot plot shows mean ± SD, each point representing **the mean enrichment ratio of aggregates for one cell**~~one aggregate~~. ***** *p* < 0.001;** **** ~~P~~ *p* < 0.0001 by Brown-Forsythe and Welch ANOVA test followed by Dunnett's multiple comparisons test.”

Fig 5 legend (p. 25) : “Dot plot shows mean ± SD, each point representing **the mean enrichment ratio of aggregates for one cell**~~one aggregate~~. **** ~~P~~ ***p*** < 0.0001 by unpaired t-test with Welch's correction.”

Fig 6 legend (p. 30) : “Dot plot shows mean ± SD, each point representing **the mean enrichment ratio of aggregates for one cell**~~one aggregate~~. ***** *p* < 0.001;** **** ~~P~~ ***p*** < 0.0001 by Brown-Forsythe and Welch ANOVA test followed by Dunnett's multiple comparisons test.”

S5 Fig legend (p. 65) : “Dot plot shows mean ± SD, each point representing **the mean enrichment ratio of aggregates for one cell.**~~one aggregate~~ ****~~P~~ ***p*** < 0. 0001 by **Kolmogorov-Smirnov test**~~unpaired t-test with Welch's correction~~.”

2. Figure S1A. What are the bands in the GFP-N lane in the Bound fraction detected with the anti-P antibody and why the intensity of the P-BFP signal is so weak in spite of considerable signal in the Input fraction? It would be important to confirm that samples have not been misassigned (e.g., potential swapping of GFP-N and P-BFP conditions).

We thank the reviewer for highlighting this discrepancy. The membrane shown in panel A was hybridised with a mixture of anti-P and anti-N antibodies. The bands in the N track correspond to degradation products of GFP-N. P-BFP capture by GFP beads is barely efficient and should not have been used. The IP experiment with GFP-P is presented in Figure 1, and the conclusions regarding the lack of Rab11a capture by P are based on this figure. Figure S1 has only been presented to enable comparison of Rab11a co-immunoprecipitation with L-GFP in the presence and absence of cross-linking, and the BFP-P and GFP-N tracks were kept as negative controls.

We repeated the immunoprecipitation experiment without crosslink. As indicated in the manuscript in the signal the signal is weak and inconsistent in absence of crosslink. We change the figure S1 accordingly.

3. Figure S2B. The conclusion that Rab11a WT and Rab11a DN exhibit distinct localization patterns (vesicular versus diffuse) is not convincingly supported by the presented images. Rab11a DN appears to display a distribution similar to WT, albeit with higher staining intensity, which complicates direct comparison. A more quantitative analysis (e.g., assessment of vesicle number, size, or distribution, or colocalization metrics) would be necessary to substantiate this claim.

The staining pattern in Figure S2 may not have been clear. We have changed the figure S2 to provide clearer images (see below). On these images, the Rab11-DN labelling appears diffuse, whereas the Rab11a WT labelling appears vesicular. This is consistent with previously published data (see, for example, doi:10.1073/pnas.95.11.6187 and doi:10.1128/JVI.00307-10). This is also clear in the larger view provided below. As Rab11a-DN is distributed uniformly throughout the cytoplasm, an analysis of vesicular labelling is not possible and colocalisation with diffuse labelling is irrelevant.

**Figure illustrating the staining pattern of Rab11a-HA wt and Rab11a-H1 DN.** A549-HA-Rab11a WT or DN cells were fixed, immunolabelled and imaged using confocal microscopy to visualize HA-Rab11a (anti-HA antibody, grey on left panel and red) and nuclei (Hoechst, blue). Scale bars: 10 μm.

Furthermore, there appears to be considerable variability in Rab11a expression levels between cells. For example, in the Rab11a DN merge panel, some cells (e.g., bottom right) show little to no detectable Rab11a signal. Such variability raises concerns about whether WT and DN conditions are directly comparable. If expression levels differ substantially, this could also impact the interpretation of downstream functional data (e.g., Fig. 8F, G). It would therefore be important to include a biochemical validation (e.g., Western blot) comparing Rab11a WT and DN expression levels.

Expression levels do vary from cell to cell, as is particularly evident in the selected image, which may therefore be misleading. We show here a larger field of view to illustrate the overall variability in Rab11a and Rab11a DN expression across the cell population. We analyzed the relative expression of Rab11 and Rab11a-DN by western blot and found no obvious global difference between the two Rab11 forms (see below). Furthermore, the lower expression of Rab11DN in the cells would result in a phenotype closer to the WT cells and could decrease the difference observed but not result in an artefactual difference.

**Figure showing the level of Rab11aHA expression in A549 cells.** A549 cells expressing either Rab11a-HAwt or Rab11a-HAS25N (DN) were lysed. Protein content was analysed by western blot revealed with anti-HA and anti-tubulin antibodies.

4. The interpretation of the MTase mutant remains unclear and, in my view, overstated. The claim that MTase activity is retained is not supported by the data. The readout used (Firefly luciferase in the minireplicon assay) reflects overall polymerase output and does not directly measure methylation. Reduced activity could equally result from impaired mRNA capping/methylation.

It has been well established that the bare polymerase activity of the L protein of RSV and related viruses (Measles, Nipah) is not sufficient to support luciferase reporter gene expression in a minigenome assay (See Dochow et al., 2012 doi: [10.1074/jbc.M111.325258](https://doi.org/10.1074/jbc.M111.325258)). We therefore consider that 25% reporter expression with our L mutant is a telltale sign that we retained at least part of the MTase activity. However, we agree that this residual activity should not be overstated and to highlight that it is partially impaired. We thus clarified the limits of our results in the corresponding results and discussion sections of the revised manuscript as follow.

Results section *Residue L1860 of polymerase L is essential for Rab11a interaction* (p. 31) : “**It has been previously shown that the MTase domain is absolutely required to produce any transcriptional activity in this assay [32], and the introduction of point mutations directly in the MTase catalytic site strongly decrease transcriptional activity [29]. Here, we confirmed that the (L_R1820A,G1855S_) mutation in catalytic residues drastically reduced the RSV transcriptional activity to ~10%, whereas the neighboring** ~~The L~~L1860A mutant retained approximately 25% of wild-type polymerase activity ~~in minigenome reporter assays~~ (S5B Fig)**. While the MTase function is altered with this mutation, it is not abrogated, we therefore evaluated the ability of the L1860A mutant to interact with Rab11a.** ~~when a L mutated in the MTase catalytic site (LR1820A,G1855S) showed only ~10% residual activity. This low however conserved activity in the minireplicon assay indicates that the MTase domain of the LL1860A is still enzymatically active.~~”

Discussion section (p. 42) : “However, the conservation of partial enzymatic activity in L demonstrates that the MTase is still functional, ~~ruling out a significant change in the conformation of this region and supporting the hypothesis that the surrounding region is involved in the interaction~~ **although its activity is significantly altered compared to wild-type. For instance, this mutation could impair the MTase domain affinity to its substrates, or even specifically impair the cap 2’O or N’7 methyltransferase activity [29]. Since the transcriptional activity is not abrogated however, we rule out a drastic change in conformation in this domain, and rather hypothesize a local structural impact in this domain that would affect the L-Rab11a interaction.**

Reviewer #2: No further issues.

Reviewer #3: My concerns are satisfied in the revised manuscript.

**Part III – Minor Issues: Editorial and Data Presentation Modifications**
 Please use this section for editorial suggestions as well as relatively minor modifications of existing data that would enhance clarity.

Reviewer #1: 5. Figure S5C. There is an error in figure referencing in the legend (Fig. 6A instead of Fig. 7A).

We thank the reviewer for pointing out this mistake. It has been corrected in the Fig. S5 legend.

Reviewer #2: (No Response)

Reviewer #3: (No Response)

**Figure resubmission:**

While revising your submission, we strongly recommend that you use PLOS’s NAAS tool ([https://ngplosjournals.pagemajik.ai/artanalysis](https://urldefense.com/v3/__https:/ngplosjournals.pagemajik.ai/artanalysis__;!!JFdNOqOXpB6UZW0!tepNVXu9mOlLKUyoxBDjbDF5Ds7PNYvqD4foqVksujQ8a87Ci92wY_rXZjeUb1aiHnYDXomnVAZIwYr95kxaRr0W1Yt3apI$)) to test your figure files. NAAS can convert your figure files to the TIFF file type and meet basic requirements (such as print size, resolution), or provide you with a report on issues that do not meet our requirements and that NAAS cannot fix.

After uploading your figures to PLOS’s NAAS tool - [https://ngplosjournals.pagemajik.ai/artanalysis](https://urldefense.com/v3/__https:/ngplosjournals.pagemajik.ai/artanalysis__;!!JFdNOqOXpB6UZW0!tepNVXu9mOlLKUyoxBDjbDF5Ds7PNYvqD4foqVksujQ8a87Ci92wY_rXZjeUb1aiHnYDXomnVAZIwYr95kxaRr0W1Yt3apI$), NAAS will process the files provided and display the results in the "Uploaded Files" section of the page as the processing is complete. If the uploaded figures meet our requirements (or NAAS is able to fix the files to meet our requirements), the figure will be marked as "fixed" above. If NAAS is unable to fix the files, a red "failed" label will appear above. When NAAS has confirmed that the figure files meet our requirements, please download the file via the download option, and include these NAAS processed figure files when submitting your revised manuscript.

**Reproducibility:**

 To enhance the reproducibility of your results, we recommend that authors of applicable studies deposit laboratory protocols in protocols.io, where a protocol can be assigned its own identifier (DOI) such that it can be cited independently in the future. Additionally, PLOS ONE offers an option to publish peer-reviewed clinical study protocols. Read more information on sharing protocols at https://plos.org/protocols?utm_medium=editorial-email&utm_source=authorletters&utm_campaign=protocols
