# Supplementary material for: Direct interaction between RSV polymerase L and active Rab11a mediates viral ribonucleoprotein transport to assembly sites
Source: PLoS Pathog. 2026 Aug 3;22(8):e1013671. doi: 10.1371/journal.ppat.1013671 (PMC13456277; doi:10.1371/journal.ppat.1013671)
Supplement: Peer review history

# Peer review: Decision Letter 0

**DOI:** 10.1371/journal.ppat.1013671.r001

**Authors:** Maria Amorim, Thomas Hoenen

10 Dec 2025

Direct interaction between RSV polymerase L and active Rab11a mediates viral ribonucleoprotein transport to assembly sites

PLOS Pathogens

Dear Dr. Rameix-Welti,

**1) Rab11a function in RSV replication**

The importance of Rab11a in the RSV replication cycle is not directly examined.

**2) Experimental and interpretative weaknesses**

The manuscript is weakened by recurring issues such as missing controls, inconsistent quantification, unclear interpretation of aggregation phenotypes, and incomplete methodological descriptions.

**3) Long-range transport and downstream consequences**

The authors conclude that vRNP long-range transport is impaired, yet they do not investigate effects on virus release or genome incorporation—processes that should also be impacted. Ideally, these experiments should be performed in Rab11a-knockout cells complemented with Rab11a-WT, CA, or DN to confirm Rab11a dependence. Moreover, if the minimal L fragment only partially competes with full-length L, one would expect some long-range trafficking events to persist; this possibility should be addressed.

**4) Crosslinking in pulldown assays**

Crosslinker is applied throughout the pulldown assays, suggesting a transient or unstable interaction, which seems inconsistent with the BLI data showing stable binding. Including non-crosslinked pulldown assays would strengthen the manuscript by revealing whether the interaction is stable under native conditions.

**5) Rab11a dominant-negative localization**

Immunofluorescence data in RSV-infected cells are needed to show whether Rab11a-DN fails to colocalize with L, directly supporting the proposed mechanism. A comparison with Rab11a-WT or CA should also be provided.

**6) Mapping the Rab11 binding site on L**

A central question is whether Rab11 binds the MTase domain, the CTD, or a site spanning both domains (Figure 5). Several sentences imply that both domains are required (e.g., line 423), though it remains likely that only one domain contains the binding site. Repeating the pulldowns with GFP-L constructs expressing only the MTase domain or only the CTD would clarify which domain is necessary for the interaction.

Please submit your revised manuscript by Feb 08 2026 11:59PM. If you will need more time than this to complete your revisions, please reply to this message or contact the journal office at plospathogens@plos.org. When you're ready to submit your revision, log on to https://www.editorialmanager.com/ppathogens/ and select the 'Submissions Needing Revision' folder to locate your manuscript file.

Please include the following items when submitting your revised manuscript:

* A rebuttal letter that responds to each point raised by the editor and reviewer(s). You should upload this letter as a separate file labeled 'Response to Reviewers'. This file does not need to include responses to any formatting updates and technical items listed in the 'Journal Requirements' section below.

* A marked-up copy of your manuscript that highlights changes made to the original version. You should upload this as a separate file labeled 'Revised Manuscript with Track Changes'.

* An unmarked version of your revised paper without tracked changes. You should upload this as a separate file labeled 'Manuscript'.

If you would like to make changes to your financial disclosure, competing interests statement, or data availability statement, please make these updates within the submission form at the time of resubmission. Guidelines for resubmitting your figure files are available below the reviewer comments at the end of this letter.

We look forward to receiving your revised manuscript.

Kind regards,

Maria João Amorim, Ph.D

Academic Editor

PLOS Pathogens

Thomas Hoenen

Section Editor

PLOS Pathogens

Editor-in-Chief

PLOS Pathogens

orcid.org/0000-0003-2946-9497

Editor-in-Chief

PLOS Pathogens

orcid.org/0000-0002-7699-2064

**Journal Requirements:**

1) We do not publish any copyright or trademark symbols that usually accompany proprietary names, eg ©,  ®, or TM  (e.g. next to drug or reagent names). Therefore please remove all instances of trademark/copyright symbols throughout the text, including:

- TM on page: 35.

2) We note that your Data Availability Statement is currently as follows: "All relevant data are within the manuscript and its Supporting Information files.". Please confirm at this time whether or not your submission contains all raw data required to replicate the results of your study. Authors must share the “minimal data set” for their submission. PLOS defines the minimal data set to consist of the data required to replicate all study findings reported in the article, as well as related metadata and methods (https://journals.plos.org/plosone/s/data-availability#loc-minimal-data-set-definition).

For example, authors should submit the following data:

1) The values behind the means, standard deviations and other measures reported;

2) The values used to build graphs;

3) The points extracted from images for analysis..

Authors do not need to submit their entire data set if only a portion of the data was used in the reported study.

If your submission does not contain these data, please either upload them as Supporting Information files or deposit them to a stable, public repository and provide us with the relevant URLs, DOIs, or accession numbers. For a list of recommended repositories, please see https://journals.plos.org/plosone/s/recommended-repositories.

If there are ethical or legal restrictions on sharing a de-identified data set, please explain them in detail (e.g., data contain potentially sensitive information, data are owned by a third-party organization, etc.) and who has imposed them (e.g., an ethics committee). Please also provide contact information for a data access committee, ethics committee, or other institutional body to which data requests may be sent. If data are owned by a third party, please indicate how others may request data access.

Note: If the reviewer comments include a recommendation to cite specific previously published works, please review and evaluate these publications to determine whether they are relevant and should be cited. There is no requirement to cite these works unless the editor has indicated otherwise.

**Reviewers' Comments:**

Reviewer's Responses to Questions

**Part I - Summary**

Please use this section to discuss strengths/weaknesses of study, novelty/significance, general execution and scholarship.

Reviewer #1: This study by Giry et al. investigates the mechanistic role of Rab11a in the intracellular trafficking of respiratory syncytial virus (RSV) viral ribonucleoprotein complexes (vRNPs). The authors propose that the viral polymerase L protein directly binds GTP-loaded Rab11a and that this interaction drives Rab11a-dependent vRNP transport. By combining co-immunoprecipitation experiments, mutagenesis, biochemical assays, and imaging analyses, the authors map the interaction interface to the Rab11a Switch I region and the C-terminal domain of L. They further suggest that this interaction is necessary for efficient vRNP trafficking to the plasma membrane and therefore for virion assembly.

Overall, this manuscript highlights a potentially important and previously underexplored mechanistic link between RSV polymerase L and Rab11a, with implications for understanding vRNP trafficking. However, the importance of Rab11a in the replication cycle of RSV is not addressed directly. Furthermore, the manuscript is weakened by recurring issues with missing controls, inconsistent quantification, unclear interpretation of aggregation phenotypes, and incomplete methodological descriptions. These concerns substantially limit the strength of the conclusions. The manuscript would also benefit from careful editorial revision to improve clarity and consistency.

Reviewer #2: General Assessment:

This manuscript by Giry et al., entitled “Direct interaction between RSV polymerase L and active Rab11a mediates viral ribonucleoprotein transport to assembly sites,” convincingly demonstrates that only GTP-bound Rab11a binds the RSV L polymerase. The study further shows that Rab11a binding does not affect polymerase activity and identifies a specific residue involved in the interaction. This work extends the findings of Cosentino et al., who demonstrated Rab11a interaction with vRNPs, and represents a well-executed and important contribution to the fields of virology and membrane trafficking.

Rab11a is a central factor in nucleocapsid trafficking across multiple RNA viruses, giving the work broad relevance. However, some conclusions rely on experiments performed only in duplicate rather than triplicate, and several conceptual gaps remain that should be addressed.

Reviewer #3: This work from Giry and colleagues describes in molecular detail the interaction between the host protein Rab11 and the RSV polymerase (L). This is a substantial body of work addressing a question of high importance in the RSV field, with significant implications for (the growing number) of other RNA viruses that also use the Rab11 pathway to traffic their genomes. The work uses a suite of orthogonal techniques to map the Rab11-RSV interaction on both the host and viral side, and extends what is known about this interaction beyond what is known for any Rab11-viral polymerase pair to date. The implications of these findings (particularly the identification of a putative Rab11 binding motif within L) are highly significant, and may lead to advances in antiviral development and further mechanistic insights for a range of Rab11-dependent RNA viruses. While the work generally employs a range of experimental strategies to prove orthogonal evidence for each conclusion (confocal/IP for instance), some discrepancies in controls need addressing (ie, equal binding of bait proteins, protein stability). The work described here has the potential to significantly advance the field, in particular if the conclusions regarding the relevant domain and point mutant in L that dictate Rab11 binding can be strengthened.

**********

**Part II – Major Issues: Key Experiments Required for Acceptance**

Please use this section to detail the key new experiments or modifications of existing experiments that should be absolutely required to validate study conclusions.

Generally, there should be no more than 3 such required experiments or major modifications for a "Major Revision" recommendation. If more than 3 experiments are necessary to validate the study conclusions, then you are encouraged to recommend "Reject".

Reviewer #1: 1. The study would be substantially strengthened by directly assessing RSV replication in Rab11a knockout cells, available from several laboratories. Alternatively, Rab11a silencing could be used. Testing the Rab11a–L association in Rab11a knockout cells, particularly for imaging, pulldown, and polymerase assays, would also help avoid confounding effects from endogenous Rab11a.

2. Many conclusions derive from overexpression that generates aggregates acknowledged by the authors as artifacts that do not occur during RSV infection. Given that availability of RSV expressing GFP-tagged L protein, it is surprising that endogenous Rab11a pulldown from infected cells was not attempted. Such data would greatly strengthen the manuscript. In addition, although the authors initially state that P is required for L stability, later pulldown assays (Fig. 5B, 5E, S5B) are performed without P. The rationale for these differing conditions should be explained.

3. Fig. 7. The authors conclude that vRNP long-range transport is impaired, yet they do not examine effects on virus release or genome incorporation, which should also be impacted. Ideally, these experiments should be repeated in Rab11a-knockout cells complemented with Rab11a-WT, CA, or DN to confirm Rab11a dependence. Furthermore, if the minimal L fragment only partially competes with full-length L, some long-range trafficking events should remain; this possibility should be addressed.

4. Loading controls (such as actin or GAPDH) and size markers are absent from multiple figures (e.g., 1A, 2A, 4B, 5E, 6A). Imaging experiments also lack untransfected cell controls for background staining.

5. Reporting of replicates is inconsistent. Several quantifications include only two biological replicates despite figure legends stating that three were performed (e.g., Fig. 1D, S4B). For pulldown quantification, intensities should be normalized to the amount of L retrieved before comparisons across conditions. This issue is particularly evident in Fig. 2A. Without proper normalization, quantification may be misleading.

6. Crosslinker is applied throughout the pulldown assays, suggesting that the interaction may be transient or unstable. This seems inconsistent with the BLI data showing stable binding. The manuscript would be strengthened by including non-crosslinked pulldown assays to assess native interaction stability.

7. It is unclear in which experiments GTP was added to activate Rab11a constructs. Even Rab11a-CA requires GTP loading, and conditions lacking supplemental GTP, such as Fig. 5G, make comparisons to prior assays difficult. A clear and consistent description of GTP usage is needed in both Methods and figure legends.

8. Fig. S6B shows that the L1860 mutation impairs polymerase activity, yet the manuscript claims retained MTase activity without direct evidence (lines 467-469). MTase assays should be included. Expression levels of L1860 relative to WT should also be shown.

Reviewer #2: Major Comments:

1. Rab11a membrane association versus membrane-less RSV inclusions (discussion)

RSV inclusion bodies containing nucleocapsid and L are membrane-less organelles. Functional Rab11 binding typically requires membrane association because the properly folded Switch I domain is stabilized on membranes. How do the authors reconcile L interaction with Rab11a under these conditions? Are nucleocapsids or inclusion bodies interacting directly or indirectly with membrane-bound Rab11a? Please expand the discussion with relevant cell biology and membrane trafficking mechanisms to propose possible models.

2. Rab11a dominant-negative localization (experiment)

The authors should provide immunofluorescence data in RSV-infected cells showing whether Rab11aDN fails to colocalize with L, which would directly support their mechanistic claims. Provide a comparison with Rab11a WT or CA.

3. Functional requirement of Rab11a for RSV replication (experiment)

Although the biochemical interaction is well supported, there remains no evidence that Rab11a function is required for productive RSV replication. Functional data would substantially strengthen the manuscript and provide definitive proof of biological relevance. The authors should provide growth kinetics of RSV in A549-Rab11a and A549-Rab11aDN cells and validate inhibition in cells expressing mCherry-L(1756–2165).

Finally, although technically challenging and possibly outside the scope of the current study, it would be valuable to determine whether a virus containing the L1860 mutation can be rescued (optional).

Reviewer #3: • A key question is whether Rab11 is binding to the MTase domain, the CTD, or a site that spans both domains (Figure 5). As currently written, many sentences describing this interaction imply that both domains are required (see line 423 for example), though I believe it is still likely that the actual binding site is in one of the two regions. Repeating the pulldowns with GFP-L constructs that express only the MTase domain or only the CTD should clarify which of the domains is required for the interaction.

• Fig 3D would fit better in a stand-alone figure focused on polymerase function- this is a key conclusion of the work, and should be highlighted as such. This should include data from the relevant supplemental figure (S3) with appropriate biological replicates. In particular, multiple replicates of the methyl transferase assay seem key here, as from the single rep shown it appears that the MTase activity decreases in the presence of the Rab11 DN construct- the lack of statistical significance could simply be that the N is too small.

• The sequence similarities between the Rab11 binding domain of FIP2 and the residues of L within the 1756-2165 portion are very intriguing. However, the fact that the L1860A mutation has such a severe effect on polymerase function complicates the interpretation of Figure 6. What if Rab11 binds only to L that is functional, and thus the loss of the Rab11-L interaction is due to the severe defect in polymerase function mediated by the L1860A mutation. Does mutation of either of the two residues immediately upstream also decrease polymerase function so dramatically? (if not, they could get around this conundrum). Would such a mutation in the MTase domain be expected to have such a dramatic effect on the minireplicon assay in any case? (vs the MT assay)- my worry is that this implies that the protein in inherently unstable. Would the MTase catalytic site mutations have a similar effect on Rab11 binding? (which could help distinguish the motif around 1860 from MTase activity?)

**********

**Part III – Minor Issues: Editorial and Data Presentation Modifications**

Please use this section for editorial suggestions as well as relatively minor modifications of existing data that would enhance clarity.

Reviewer #1: In the abstract, the phrase “its involvement” is ambiguous and should be clarified.

In the introduction, it is unclear whether “ubiquitously expressed” refers to Rab11a, Rab11b, or both.

Fig. 1A: BFP-P shows multiple bands; please address.

Fig. 3: SEC profiles and purification gels for L+P should be shown.

Fig. 3D: Clarify why two vehicle controls are included and explain the normalization strategy. Which bands were included in the quantification?

Reviewer #2: Minor / Technical Comments:

1. Crosslinking and Western blot analysis

• Please explain the presence of three P-BFP bands.

• Does the anti-GFP antibody recognize GFP and BFP with equal affinity?

• Include L-only controls for co-IP western blots and IF to confirm specificity. Since L stability depends on P, L alone should not bind.

• Figure 1 appears to show only duplicate experiments. Please provide results from at least three independent replicates.

2. L truncation mapping

Please provide evidence that the truncated L constructs used for interaction mapping are properly folded. Loss of binding could result from misfolding rather than loss of the Rab11-interaction interface, limiting interpretation of negative results.

3. Figure 5G (GST pulldown)

• Why does GFP-L(1756–2165) migrate as two bands?

• Why is binding to Rab11a WT weak?

• Was GTP included in the assay?

4. L1860 mutation

Please clarify the rationale for selecting L1860, especially as this residue does not reside within the GxGxG motif initially implicated. Why were not all glycines mutated initially? Were additional residues tested? Is this motif surface-exposed in the L structure? Please provide a structural figure highlighting the mutation site.

5. L inclusion formation

Does L form cytoplasmic inclusions in the absence of GFP tagging?

Reviewer #3: • BFP doesn’t bind as well to GFP trap (both in theory, and in Fig 1A where the efficiency of P-BFP pulldown vs the GFP pulldowns is very low. This makes it hard to completely rule out Rab11 binding to P (or a joint P/L epitope).

• In Figure 1C the area chosen for line intensity scans are not easy to compare. The L-GFP+P condition has two distinct red peaks, representing discrete Rab11 positive structures. While I appreciate that the addition of L-GFP+P relocalizes Rab11 into multiple cytoplasmic structures, it would be more helpful for comparison to do the line intensity scans over the perinuclear Rab11 compartment (or another place in the image where there is a red peak of similar size/separation from background signal).

• While the data in figure 2B argue convincingly that the DN version of Rab11 neither binds to L or induces its relocalization upon co-transfection, it is hard to make conclusions about the CA construct (Fig 2B). The efficiency of pulldown for L-GFP appears ~50% more efficient in the condition where it was co-transfected with Rab11 CA (vs the transfection with WT)- based on the L-GFP ‘bound’ lanes. While there is less HA-Rab11a in the ‘WT’ condition, there is less GFP-polymerase there as well, making it possible that the increased binding for CA is just reflective of more bound bait.

• As in Fig 2B, the amount of L-GFP that is pulled down in the IGV and AAA conditions do not appear equal, which complicates the interpretation of this experiment. There is significantly more L-GFP present in the ‘Bound IGV’ sample vs the ‘Bound AAA’ sample, despite equivalent inputs, which raises the possibility that the differences in HA-Rab11 binding to L are the result of variations in binding efficiency. Multiple biological replicates with equal binding are needed to sort this out. In addition, it is difficult to assess changes in colocalization (Fig 4C), it would help to display single channel images of the zoomed in areas. But from the lower mag images it appears that the L-GFP is still found in areas of Rab11 concentration with the AAA construct (see perinuclear structure in lower left cell, large aggregate in upper right cell that appear enriched in both channels). Also, the quantification in Figure S4 should be moved into the main figure.

**********

PLOS authors have the option to publish the peer review history of their article (what does this mean?). If published, this will include your full peer review and any attached files.

If you choose “no”, your identity will remain anonymous but your review may still be made public.

**Do you want your identity to be public for this peer review?**  For information about this choice, including consent withdrawal, please see our Privacy Policy.

Reviewer #1: No

Reviewer #2: No

Reviewer #3: No

[NOTE: If reviewer comments were submitted as an attachment file, they will be attached to this email and accessible via the submission site. Please log into your account, locate the manuscript record, and check for the action link "View Attachments". If this link does not appear, there are no attachment files.]

**Figure resubmission:**

**Reproducibility:**

?>

# Author comment: Author response to Decision Letter 1

**DOI:** 10.1371/journal.ppat.1013671.r002

9 Apr 2026

Attachment Submitted filename: Response to Reviewers.docx

# Peer review: Decision Letter 1

**DOI:** 10.1371/journal.ppat.1013671.r003

**Authors:** Maria Amorim, Thomas Hoenen

4 May 2026

PPATHOGENS-D-25-02679R1

Direct interaction between RSV polymerase L and active Rab11a mediates viral ribonucleoprotein transport to assembly sites

PLOS Pathogens

Dear Dr. Rameix-Welti,

Thank you for submitting your manuscript to PLOS Pathogens. After careful consideration, we feel that it has merit but does not fully meet PLOS Pathogens's publication criteria as it currently stands. Therefore, we invite you to submit a revised version of the manuscript that addresses the points raised during the review process.

Please submit your revised manuscript by Jul 03 2026 11:59PM. If you will need more time than this to complete your revisions, please reply to this message or contact the journal office at plospathogens@plos.org. When you're ready to submit your revision, log on to https://www.editorialmanager.com/ppathogens/ and select the 'Submissions Needing Revision' folder to locate your manuscript file.

Please include the following items when submitting your revised manuscript:

* A letter that responds to each point raised by the editor and reviewer(s). You should upload this letter as a separate file labeled 'Response to Reviewers'. This file does not need to include responses to any formatting updates and technical items listed in the 'Journal Requirements' section below.

* A marked-up copy of your manuscript that highlights changes made to the original version. You should upload this as a separate file labeled 'Revised Manuscript with Track Changes'.

* An unmarked version of your revised paper without tracked changes. You should upload this as a separate file labeled 'Manuscript'.

If you would like to make changes to your financial disclosure, competing interests statement, or data availability statement, please make these updates within the submission form at the time of resubmission. Guidelines for resubmitting your figure files are available below the reviewer comments at the end of this letter.

We look forward to receiving your revised manuscript.

Kind regards,

Maria João Amorim, Ph.D

Academic Editor

PLOS Pathogens

Thomas Hoenen

Section Editor

PLOS Pathogens

Sumita Bhaduri-McIntosh

Editor-in-Chief

PLOS Pathogens

orcid.org/0000-0003-2946-9497

Michael Malim

Editor-in-Chief

PLOS Pathogens

orcid.org/0000-0002-7699-2064

**Additional Editor Comments:**

The reviewers have now completed their assessment of the revised version of your manuscript. Two reviewers are very positive about the improvements made, while Reviewer 1 has raised several important points that would benefit from further clarification before a final decision can be reached.

We understand that additional revisions may extend the timeline, and we appreciate the effort this entails. While we are not in a position to guarantee acceptance at this stage, the supportive evaluations from the other reviewers suggest that the manuscript is progressing well. We therefore encourage you to consider addressing the remaining comments, as this would strengthen the manuscript and support its further consideration in the journal.

**Reviewers' Comments:**

Reviewer's Responses to Questions

**Part I - Summary**

Please use this section to discuss strengths/weaknesses of study, novelty/significance, general execution and scholarship.

Reviewer #1: In this revised version, the authors aim to further develop their mechanistic model in which RSV polymerase L directly interacts with GTP-bound Rab11a to mediate vRNP transport. The manuscript has improved in several aspects, including the addition of replication kinetics and further pulldown experiments. However, some of the concerns raised previously do not appear to be fully resolved and the new data are also somewhat problematic.

Overall, the study addresses an important question and has clear potential. Nevertheless, in its current form, some issues remain that limit the strength of the conclusions and would benefit from additional clarification or experimental support.

Reviewer #2: The authors have addressed all my comments and provided the required experiements where ever it was possible. It is understandable that rescuing the L1860 virus and performing RSV growth kinetics in cells expressing mCherry-L1756-2165 is technically challenging and can be completed in future studies.

Reviewer #3: (No Response)

**********

**Part II – Major Issues: Key Experiments Required for Acceptance**

Please use this section to detail the key new experiments or modifications of existing experiments that should be absolutely required to validate study conclusions.

Generally, there should be no more than 3 such required experiments or major modifications for a "Major Revision" recommendation. If more than 3 experiments are necessary to validate the study conclusions, then you are encouraged to recommend "Reject".

Reviewer #1: 1. The statistical analysis remains problematic. The authors now report only two biological replicates, yet still obtain extremely low p-values (e.g., p < 0.0001) (Figure 1D, Figure 6C, D). This is not plausible given such low replicate numbers and suggests that individual cells may have been pooled and treated as independent observations. If so, this would constitute inappropriate statistical treatment and artificially inflate significance.

2. Figure S1A. What are the bands in the GFP-N lane in the Bound fraction detected with the anti-P antibody and why the intensity of the P-BFP signal is so weak in spite of considerable signal in the Input fraction? It would be important to confirm that samples have not been misassigned (e.g., potential swapping of GFP-N and P-BFP conditions).

3. Figure S2B. The conclusion that Rab11a WT and Rab11a DN exhibit distinct localization patterns (vesicular versus diffuse) is not convincingly supported by the presented images. Rab11a DN appears to display a distribution similar to WT, albeit with higher staining intensity, which complicates direct comparison. A more quantitative analysis (e.g., assessment of vesicle number, size, or distribution, or colocalization metrics) would be necessary to substantiate this claim. Furthermore, there appears to be considerable variability in Rab11a expression levels between cells. For example, in the Rab11a DN merge panel, some cells (e.g., bottom right) show little to no detectable Rab11a signal. Such variability raises concerns about whether WT and DN conditions are directly comparable. If expression levels differ substantially, this could also impact the interpretation of downstream functional data (e.g., Fig. 8F, G). It would therefore be important to include a biochemical validation (e.g., Western blot) comparing Rab11a WT and DN expression levels.

4. The interpretation of the MTase mutant remains unclear and, in my view, overstated. The claim that MTase activity is retained is not supported by the data. The readout used (Firefly luciferase in the minireplicon assay) reflects overall polymerase output and does not directly measure methylation. Reduced activity could equally result from impaired mRNA capping/methylation.

Reviewer #2: No further issues.

Reviewer #3: My concerns are satisfied in the revised manuscript.

**********

**Part III – Minor Issues: Editorial and Data Presentation Modifications**

Please use this section for editorial suggestions as well as relatively minor modifications of existing data that would enhance clarity.

Reviewer #1: 5. Figure S5C. There is an error in figure referencing in the legend (Fig. 6A instead of Fig. 7A).

Reviewer #2: (No Response)

Reviewer #3: (No Response)

**********

PLOS authors have the option to publish the peer review history of their article (what does this mean?). If published, this will include your full peer review and any attached files.

If you choose “no”, your identity will remain anonymous but your review may still be made public.

**Do you want your identity to be public for this peer review?**  For information about this choice, including consent withdrawal, please see our Privacy Policy.

Reviewer #1: No

Reviewer #2: No

Reviewer #3: No

[NOTE: If reviewer comments were submitted as an attachment file, they will be attached to this email and accessible via the submission site. Please log into your account, locate the manuscript record, and check for the action link "View Attachments". If this link does not appear, there are no attachment files.]

**Figure resubmission:**

**Reproducibility:**

To enhance the reproducibility of your results, we recommend that authors of applicable studies deposit laboratory protocols in protocols.io, where a protocol can be assigned its own identifier (DOI) such that it can be cited independently in the future. Additionally, PLOS ONE offers an option to publish peer-reviewed clinical study protocols. Read more information on sharing protocols at https://plos.org/protocols?utm_medium=editorial-email&utm_source=authorletters&utm_campaign=protocols

# Author comment: Author response to Decision Letter 2

**DOI:** 10.1371/journal.ppat.1013671.r004

17 Jun 2026

Attachment Submitted filename: Response to Reviewers - Rev2.docx

# Editor report: Decision Letter 2

**DOI:** 10.1371/journal.ppat.1013671.r005

**Authors:** Maria Amorim, Thomas Hoenen

29 Jun 2026

PPATHOGENS-D-25-02679R2

Direct interaction between RSV polymerase L and active Rab11a mediates viral ribonucleoprotein transport to assembly sites

PLOS Pathogens

Dear Dr. Rameix-Welti,

Thank you for submitting your manuscript to PLOS Pathogens. After careful consideration, we feel that it has merit but does not fully meet PLOS Pathogens's publication criteria as it currently stands. Therefore, we invite you to submit a revised version of the manuscript that addresses the points raised during the review process.

Please submit your revised manuscript by Aug 28 2026 11:59PM. If you will need more time than this to complete your revisions, please reply to this message or contact the journal office at plospathogens@plos.org. When you're ready to submit your revision, log on to https://www.editorialmanager.com/ppathogens/ and select the 'Submissions Needing Revision' folder to locate your manuscript file.

Please include the following items when submitting your revised manuscript:

* A letter that responds to each point raised by the editor and reviewer(s). You should upload this letter as a separate file labeled 'Response to Reviewers'. This file does not need to include responses to any formatting updates and technical items listed in the 'Journal Requirements' section below.

* A marked-up copy of your manuscript that highlights changes made to the original version. You should upload this as a separate file labeled 'Revised Manuscript with Track Changes'.

* An unmarked version of your revised paper without tracked changes. You should upload this as a separate file labeled 'Manuscript'.

If you would like to make changes to your financial disclosure, competing interests statement, or data availability statement, please make these updates within the submission form at the time of resubmission. Guidelines for resubmitting your figure files are available below the reviewer comments at the end of this letter.

As the corresponding author, your ORCID iD is verified in the submission system and will appear in the published article. PLOS supports the use of ORCID, and we encourage all coauthors to register for an ORCID iD and use it as well. Please encourage your coauthors to verify their ORCID iD within the submission system before final acceptance, as unverified ORCID iDs will not appear in the published article. Only the individual author can complete the verification step; PLOS staff cannot verify ORCID iDs on behalf of authors.

We look forward to receiving your revised manuscript.

Kind regards,

Maria João Amorim, Ph.D

Academic Editor

PLOS Pathogens

Thomas Hoenen

Section Editor

PLOS Pathogens

Sumita Bhaduri-McIntosh

Editor-in-Chief

PLOS Pathogens

orcid.org/0000-0003-2946-9497

Michael Malim

Editor-in-Chief

PLOS Pathogens

orcid.org/0000-0002-7699-2064

**Additional Editor Comments (if provided):**

Dear Dr. Rameix-Welti,

Thank you for submitting your revised manuscript to *PLOS Pathogens*. The editorial team has carefully evaluated the revised version and agrees that the manuscript has been substantially improved following the reviewers' comments.

However, the editorial team would like to raise one additional point that was not addressed during peer review. Despite being an unusual request, the editorial team considers that an answer to these concerns would substantially improve the overall understanding of role of Rab11a in RSV infection. We hope the authors agree with us.

In Figures 1A and 1C, the different viral proteins are analysed under non-equivalent experimental conditions. For consistency, these conditions should be normalized. Specifically, we believe it is important to repeat the experiments in Figure 3 to include the P+L, P+N, P+M2-1 and P+M conditions to demonstrate that Rab11a does not relocalize to the condensates (or other structures) formed under these conditions. This control is particularly important because P+N and P+M2-1 have previously been shown to undergo phase separation, and the present study suggests that P+L may also form condensates/aggregates. It is therefore important to distinguish whether Rab11a recruitment is specific to the L-containing complex or simply a consequence of condensate formation.

In addition, please clarify why the manuscript refers to the P+L structures as "aggregates" rather than phase-separated condensates.

Finally, the Discussion currently suggests that the P+L aggregates are likely to be an artifact. However, the fact that they do not happen in infection does not mean that they may not happen in this transfection system. A plausible alternative explanation is that they arise from the experimental conditions, such as the high protein concentrations achieved in the transient transfection system, which may not faithfully mimic viral infection or that somehow N restricts the entrance of Rab11a into IB from RSV infected cells. Please comment on this possibility.

**Minor issues**

1. For clarity, lines 146–147 state: "To identify the viral protein that mediates vRNP-Rab11a binding, we performed co-IP experiments using HEK293T cells transiently co-expressing HA-Rab11a and one of the vRNP proteins (N, P, L or M2-1) fused to GFP." However, Figure 1A does not include an L-GFP condition alone. Please revise the text to indicate that L was co-expressed with P. In addition, please move the subsequent sentence (lines 156–157), "Although GFP-L was co-expressed with P to ensure its stability, Rab11a specifically co-immunoprecipitated with the L-P complex but not with P alone, indicating that L mediates the interaction with Rab11a," immediately after the introductory description of the experimental design.

2. The same issue applies to Figure 1C, where no L-GFP-alone condition is shown. Please revise the corresponding text accordingly.

3. In Figure 1C, the Rab11a staining in the P-alone condition does not resemble the expected Rab11a localization and appears inconsistent with the other panels. Please verify that the correct image has been included.

4. In Figure 3A, do the authors have an explanation for why the measured Kd is approximately four-fold lower (i.e., the affinity is higher) for HA-Rab11a WT? One would expect the affinities to be broadly similar or, if anything, for the constitutively active Rab11a mutant to exhibit the higher affinity. Such higher affinity of the Rab11CA construct is apparent in Figure 6E. In addition, could the authors include immunofluorescence images showing co-expression of the P(CTD) construct and L? This would be very useful

5. Please correct the L-GFP+P panel in Figure 5D.

6. In Figure 6B, the immunofluorescence images do not convincingly demonstrate co-localization between any of the L domains and Rab11a. Instead, the L fragments appear largely diffuse throughout the nucleus and cytoplasm without obvious enrichment at Rab11a-positive structures. a possibility is to change this panel to the supplementary material because it does not add information to the figure.

8. In line 197, please capitalize "HA-Rab11a." The sentence should read: "The indicated viral protein was expressed alone (left column) or together with HA-Rab11a."

**Reviewers' Comments:**

[NOTE: If reviewer comments were submitted as an attachment file, they will be attached to this email and accessible via the submission site. Please log into your account, locate the manuscript record, and check for the action link "View Attachments". If this link does not appear, there are no attachment files.]

**Figure resubmission:**

**Reproducibility:**

To enhance the reproducibility of your results, we recommend that authors of applicable studies deposit laboratory protocols in protocols.io, where a protocol can be assigned its own identifier (DOI) such that it can be cited independently in the future. Additionally, PLOS ONE offers an option to publish peer-reviewed clinical study protocols. Read more information on sharing protocols at https://plos.org/protocols?utm_medium=editorial-email&utm_source=authorletters&utm_campaign=protocols

# Author comment: Author response to Decision Letter 3

**DOI:** 10.1371/journal.ppat.1013671.r006

6 Jul 2026

Attachment Submitted filename: Response to Editorial team.pdf

# Editor report: Decision Letter 3

**DOI:** 10.1371/journal.ppat.1013671.r007

**Authors:** Maria Amorim, Thomas Hoenen

9 Jul 2026

Dear Pr Rameix-Welti,

We are pleased to inform you that your manuscript 'Direct interaction between RSV polymerase L and active Rab11a mediates viral ribonucleoprotein transport to assembly sites' has been provisionally accepted for publication in PLOS Pathogens.

Before your manuscript can be formally accepted you will need to complete some formatting changes, which you will receive in a follow up email. A member of our team will be in touch with a set of requests.

Please note that your manuscript will not be scheduled for publication until you have made the required changes, so a swift response is appreciated.

IMPORTANT: The editorial review process is now complete. PLOS will only permit corrections to spelling, formatting or significant scientific errors from this point onwards. Requests for major changes, or any which affect the scientific understanding of your work, will cause delays to the publication date of your manuscript. However, as a note, we thank you for your resilience in the review process. The IF figure you included in the last rebuttal letter with Rab11 recruitment in the presence of L+P, N+P, P+M2-1 is beautiful and serves perfectly the point that it is not P or P phase separating that recruits Rab11a. The editorial team thinks that its inclusion as an additional supplementary figure would benefit the understanding of the paper and would serve completely to eliminate the discrepancies in the conditions of the IF and pull down. We leave up to the authors if you would like to include it in the manuscript, even if facing delays.

Should you, your institution's press office or the journal office choose to press release your paper, you will automatically be opted out of early publication. We ask that you notify us now if you or your institution is planning to press release the article. All press must be co-ordinated with PLOS.

Thank you again for supporting Open Access publishing; we are looking forward to publishing your work in PLOS Pathogens.

Best regards,

Maria João Amorim, Ph.D

Academic Editor

PLOS Pathogens

Thomas Hoenen

Section Editor

PLOS Pathogens

Sumita Bhaduri-McIntosh

Editor-in-Chief

PLOS Pathogens

orcid.org/0000-0003-2946-9497

Michael Malim

Editor-in-Chief

PLOS Pathogens

orcid.org/0000-0002-7699-2064

***********************************************************

Reviewer Comments (if any, and for reference):

# Editor report: Acceptance letter

**DOI:** 10.1371/journal.ppat.1013671.r008

**Authors:** Maria Amorim, Thomas Hoenen

Dear Pr Rameix-Welti,

We are delighted to inform you that your manuscript, "Direct interaction between RSV polymerase L and active Rab11a mediates viral ribonucleoprotein transport to assembly sites," has been formally accepted for publication in PLOS Pathogens.

We have now passed your article onto the PLOS Production Department who will complete the rest of the pre-publication process. All authors will receive a confirmation email upon publication.

The corresponding author will soon be receiving a typeset proof for review, to ensure errors have not been introduced during production. Please review the PDF proof of your manuscript carefully, as this is the last chance to correct any scientific or type-setting errors. Please note that major changes, or those which affect the scientific understanding of the work, will likely cause delays to the publication date of your manuscript. Note: Proofs for Front Matter articles (Pearls, Reviews, Opinions, etc...) are generated on a different schedule and may not be made available as quickly.

Soon after your final files are uploaded, the early version of your manuscript, if you opted to have an early version of your article, will be published online. The date of the early version will be your article's publication date. The final article will be published to the same URL, and all versions of the paper will be accessible to readers.

For Research Articles, you will receive an invoice from PLOS for your publication fee after your manuscript has reached the completed accept phase. If you receive an email requesting payment before acceptance or for any other service, this may be a phishing scheme. Learn how to identify phishing emails and protect your accounts at https://explore.plos.org/phishing.

Thank you again for supporting open-access publishing; we are looking forward to publishing your work in PLOS Pathogens.

Best regards,

Sumita Bhaduri-McIntosh

Editor-in-Chief

PLOS Pathogens

orcid.org/0000-0003-2946-9497

Michael Malim

Editor-in-Chief

PLOS Pathogens

orcid.org/0000-0002-7699-2064
